# Association between neutrophile-to-lymphocyte ratio and risk of deep vein thrombosis in patient receiving lower extremity orthopedic surgery: A meta-analysis

**DOI:** 10.1371/journal.pone.0319107

**Published:** 2025-02-24

**Authors:** I-Wen Chen, Wei-Ting Wang, Kuo-Chuan Hung

**Affiliations:** 1 Department of Anesthesiology, Chi Mei Medical Center, Liouying, Tainan City, Taiwan; 2 Department of Anesthesiology, E-Da Hospital, I-Shou University, Kaohsiung, Taiwan; 3 Department of Anesthesiology, Chi Mei Medical Center, Tainan City, Taiwan; Ataturk University Faculty of Medicine, TÜRKIYE

## Abstract

**Objective:**

This meta-analysis aimed to quantitatively synthesize evidence on the association between the preoperative neutrophil-to-lymphocyte ratio (NLR) and the risk of deep vein thrombosis (DVT) in patients undergoing lower extremity orthopedic surgery.

**Methods:**

Medline, Embase, Google Scholar, and the Cochrane Library were systematically searched for observational studies that examined the relationship between preoperative NLR and DVT risk in patients undergoing lower extremity procedures. Pooled odds ratios (ORs) with 95% confidence intervals (CIs) were calculated using random-effects models.

**Results:**

Ten studies involving 5,697 patients were included in the meta-analysis. The pooled incidence of DVT across the studies was 13.8% (95% CI: 9.7%–19.2%). Elevated NLR was associated with a two-fold increase in the risk of DVT (odds ratio [OR] 2.08, 95% confidence interval [CI]: 1.39–3.12, p = 0.0004, I^2^ = 85%). Patients who developed DVT had a higher NLR than those who did not (mean difference: 0.93, 95% CI: 0.37 to 1.48, p =  0.001, I^2^ =  86%). Among the patient characteristics, male sex (OR 1.51, 95% CI: 1.12–2.03), diabetes mellitus (OR 1.60, 95% CI: 1.06–2.41), and hypertension (OR 1.43, 95% CI: 1.06–1.93) were significantly associated with increased DVT risk. Subgroup analysis based on the timing of DVT occurrence (preoperative vs. postoperative) revealed no significant difference in the association between NLR and DVT risk.

**Conclusion:**

Elevated preoperative NLR was significantly associated with increased DVT risk in patients undergoing lower extremity orthopedic surgery. NLR may serve as a useful biomarker for DVT risk stratification in this population. Prospective studies are needed to validate its predictive value and evaluate NLR-guided thromboprophylaxis strategies.

**Trial registration:**

PROSPERO registration number: CRD42024577952.

## 1. Introduction

Deep vein thrombosis (DVT) is a common and potentially life-threatening complication that often arises after lower extremity orthopedic surgery [1–4]. The incidence of DVT following such procedures ranges between 27.3% and 38.4% [5–7]. Importantly, DVT can also develop preoperatively in patients scheduled for lower-limb surgery [8], with reported incidence rates ranging from 24.1% to 32% [9,10]. This preoperative occurrence of DVT is particularly concerning, as it can complicate surgical planning and increase the risk of further complications during and after surgery. The presence of DVT in hospitalized patients is associated with significant morbidity and mortality, leading to prolonged hospital stays, increased risk of readmission, and potentially fatal complications such as pulmonary embolism [11,12]. Given the high incidence of DVT, it is crucial to identify preoperative risk factors in patients undergoing lower extremity orthopedic surgery. Accurate risk assessment and early detection of high-risk patients are vital to guide appropriate prophylactic measures and management strategies. While clinical factors such as advanced age, diabetes, hypertension, a longer time from injury to surgery, and prior thromboembolic event [13–15] are well-established risk factors, there is an increasing interest in the predictive value of biomarkers, particularly inflammatory markers, for assessing DVT risk [16–20].

Inflammation and coagulation are intricately connected, with numerous overlapping molecular pathways and bidirectional communication between the two systems [21]. This interplay contributes to a heightened risk of DVT in individuals with both acute and chronic inflammatory conditions [22–26]. The neutrophil-to-lymphocyte ratio (NLR) is a simple, cost-effective, and widely available marker of systemic inflammation. An elevated NLR has been associated with an increased risk of DVT in various clinical settings, including cancer and COVID-19 infection [27–31]. Consistently, a recent meta-analysis reported that NLR had moderate diagnostic accuracy and could serve as a useful biomarker for diagnosing DVT [32]. However, the previous meta-analysis, which included 11 studies, had only three that specifically focused on patients undergoing lower extremity orthopedic surgery [32]. As a result, the heterogeneity in the patient populations in that meta-analysis [32] may limit the applicability of the findings for providing precise clinical guidance for patients undergoing surgery on the lower extremities. To provide a comprehensive understanding of the association between NLR and DVT risk in patients undergoing orthopedic surgery for the lower extremities, we conducted a meta-analysis of available studies. The primary objective of this meta-analysis was to quantitatively synthesize evidence on the association between preoperative NLR and the risk of DVT in patients undergoing lower extremity orthopedic surgery. Secondary outcomes included assessing the pooled incidence of DVT, exploring the relationship between patient characteristics and comorbidities with DVT risk, and comparing the mean preoperative NLR between patients who developed DVT and those who did not.

## 2. Method

### 2.1. Protocol registration

This meta-analysis adhered to the Preferred Reporting Items for Systematic Reviews and Meta-Analyses (PRISMA) guidelines to ensure a comprehensive and transparent approach (S1 Table). To further enhance transparency and reduce the risk of reporting bias, the study protocol was registered with the International Prospective Register of Systematic Reviews (PROSPERO) under registration number CRD42024577952.

### 2.2. Search strategy

A thorough literature search was performed to identify studies examining the link between NLR and the risk of DVT in patients undergoing lower extremity orthopedic surgery. The search included the electronic databases Medline, Embase, Google Scholar, and Cochrane Library, covering all records up to August 2024. The search strategy utilized a mix of keywords and Medical Subject Headings (MeSH) terms related to NLR, DVT, and lower extremity orthopedic surgery. The search terms used were: (“neutrophil-to-lymphocyte ratio” OR “neutrophil lymphocyte ratio” OR “neutrophil/lymphocyte ratio” OR NLR) AND (“venous thromboembolism” OR “deep vein thrombosis” OR VTE OR DVT OR “venous thrombosis” OR “thrombotic event”) AND (“lower extremity surgery” OR “hip arthroplasty” OR “knee arthroplasty” OR “hip replacement” OR “knee replacement” OR THA OR TKA OR “joint arthroplasty” OR “joint replacement” OR “orthopedic surgery” OR “orthopaedic surgery”). The search was not restricted by language and focused on peer-reviewed studies. Additionally, the reference lists of the included studies and pertinent review articles were manually reviewed to identify any other eligible studies.

Two reviewers (I.-W.C. and W.-T.W.) independently conducted the literature search and screened the titles and abstracts of the retrieved articles. The full texts of potentially relevant studies were reviewed to determine eligibility based on predefined inclusion and exclusion criteria. Discrepancies between reviewers were resolved through discussion or by consulting with a third reviewer (K.-C.H.). Detailed search strategies for one of the databases (i.e., Medline) are provided in Table 1.

**Table 1:** Search strategy for medline.

1	(“neutrophil-to-lymphocyte ratio” or “neutrophil lymphocyte ratio” or “neutrophil/lymphocyte ratio” or NLR).mp.
2	(“venous thromboembolism” or “deep vein thrombosis” or VTE or DVT or “venous thrombosis” or “thrombotic event”).mp.
3	exp “Venous Thrombosis”/
4	(“lower extremity orthopedic surgery” or “hip arthroplasty” or “knee arthroplasty” or “hip replacement” or “knee replacement” or THA or TKA or “joint arthroplasty” or “joint replacement” or “orthopedic surgery” or “orthopaedic surgery”).mp.
5	exp “Arthroplasty, Replacement, Knee”/ or exp “Arthroplasty, Replacement, Hip”/ or exp “Orthopedic Procedures”/
6	1 and (2 or 3) and (4 or 5)

### 2.3. Inclusion and exclusion criteria

To be included in this meta-analysis, studies had to meet the following criteria: (1) population: patients undergoing lower extremity surgeries, such as total hip arthroplasty; (2) exposure: patients with elevated NLR before surgery; (3) control: patients without elevated NLR before surgery; and (4) outcomes: the occurrence of preoperative or postoperative DVT diagnosed using objective methods, such as ultrasound, venography, or computed tomography. Additionally, only observational studies (cohort, case-control, or cross-sectional) published as full-text articles in peer-reviewed journals were included.

Studies were excluded based on the following criteria: (1) case series, reviews, editorials, or conference abstracts; (2) studies that did not report the association between NLR and DVT risk or lacked sufficient data to calculate effect estimates; and (3) animal studies or in vitro experiments. Two reviewers (I.-W.C. and W.-T.W.) independently evaluated the eligibility of studies according to these criteria.

### 2.4. Outcome

The primary outcome was to determine the relationship between preoperative NLR and DVT risk in patients undergoing lower extremity orthopedic surgery, expressed as odds ratios (ORs) with 95% confidence intervals (CIs). Secondary outcomes included assessing the pooled incidence of DVT, exploring the relationship between patient characteristics (such as age and sex) and comorbidities (such as hypertension) with DVT risk, and comparing the mean preoperative NLR between patients who developed DVT and those who did not.

### 2.5. Data collection

Two reviewers (I.-W.C. and W.-T.W.) independently extracted data from the included studies using a standardized data collection form. The extracted information included the first author, publication year, country, sample size, patient characteristics (age, sex, and type of surgery), country, NLR cut-off values, and diagnostic methods for DVT. If the required data were not directly reported in the studies, they were calculated from the available information or were obtained by contacting the original author. Any discrepancies in the extracted data were resolved through discussion or consultation with a third reviewer (K.-C.H.).

### 2.6. Quality of study

The quality of the included studies was independently evaluated by two reviewers (I.-W.C. and W.-T.W.) using the Newcastle-Ottawa Scale (NOS) for observational studies. The NOS assesses study quality across three domains: selection, comparability, and outcome. Each study received a maximum of nine points, with higher scores indicating superior methodological quality. Studies scoring 7 or more were considered high quality, those scoring 5 to 6 were deemed moderate quality, and those scoring 4 or less were classified as low quality.

### 2.7. Certainty of evidence

The certainty of evidence for each outcome was evaluated using the Grading of Recommendations, Assessment, Development, and Evaluation (GRADE) approach, which systematically assesses the quality of evidence and strength of recommendations in systematic reviews. Initially rated as low for observational studies, the certainty of evidence was subsequently downgraded based on criteria such as the risk of bias (due to methodological limitations), inconsistency (unexplained variability across studies), indirectness (relevance to the review question and target population), imprecision (based on the width of confidence intervals), and potential publication bias (selective reporting). Each outcome was assigned a certainty rating of high, moderate, low, or very low. The assessments were conducted independently by two reviewers (I.-W.C. and W.-T.W.), and disagreements were resolved through discussion or consultation with a third reviewer (K.-C.H.).

### 2.8. Statistical analysis

A meta-analysis was conducted using the inverse variance method with random-effects models to compute the pooled odds ratios (ORs) and 95% confidence intervals (CIs) for the association between NLR and DVT risk. For studies that reported NLR as a categorical variable, effect estimates for the highest versus lowest categories (e.g., Q4 vs. Q1) were used. Heterogeneity among the studies was assessed using the I² statistic, with I² values of 25%, 50%, and 75% indicating low, moderate, and high heterogeneity, respectively. Subgroup analyses were performed based on the timing of DVT occurrence (i.e., preoperative or postoperative) to identify potential sources of heterogeneity. Sensitivity analyses were conducted using the leave-one-out approach, where one study at a time was removed and the pooled estimates were recalculated to test result robustness. Publication bias was assessed using funnel plots and Egger’s test, with P <  0.05 indicating significant bias. To address the issue of heterogeneity and further explore the potential impact of age on NLR-DVT association, we conducted a meta-regression analysis based on the age of the study population. All statistical analyses were performed using RevMan software (version 5.4, The Cochrane Collaboration) and Comprehensive Meta-Analysis (version 4, Biostat, Englewood, NJ, USA).

## 3. Results

### 3.1. Study selection

**Fig 1:**
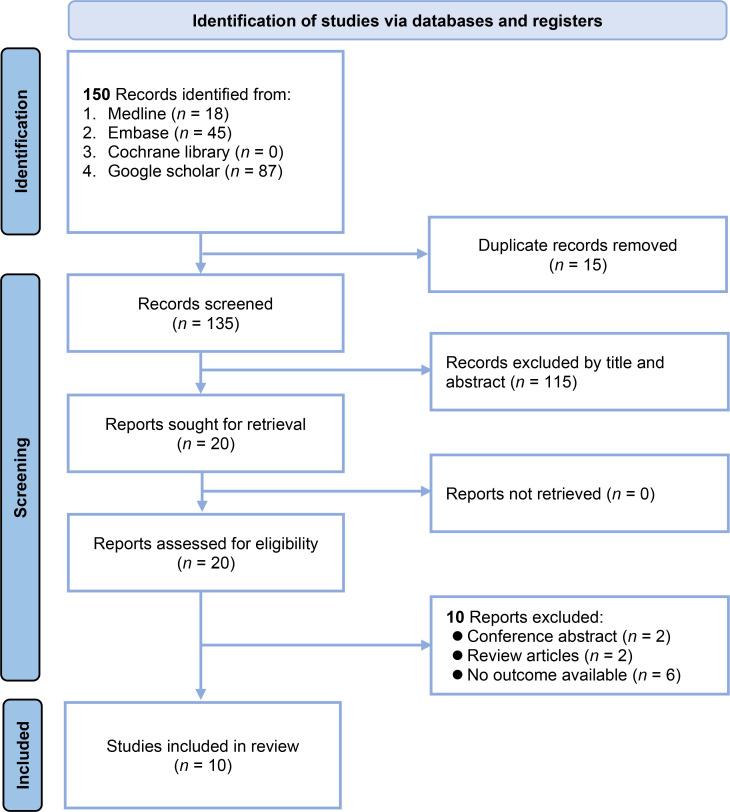
Flow chart of study selection.

The study selection process is summarized in Fig 1. The database search identified 150 records. After the removal of 15 duplicate records, 135 records were screened by title and abstract. Of these, 115 records were excluded because they did not meet the inclusion criteria based on the title and abstract review. The full texts of the remaining 20 reports were sought for retrieval and further assessment of eligibility. Upon review, 10 reports were excluded for the following reasons: two were conference abstracts, two were review articles, and six had no relevant outcome data available ( S3 Table). In total, 10 studies met the inclusion criteria and were included in this systematic review and meta-analysis [16,33–41].

### 3.2. Characteristics of studies

The main characteristics of the 10 studies included in this meta-analysis are summarized in Table 2. These studies were published between 2018 and 2023. The sample sizes ranged from 104 to 1179 patients, with 5697 patients across all studies. The mean age of participants ranged from 42.8 to 83 years, and the percentage of male participants varied from 10% to 62.9%. Eight studies were conducted in China [16,33,34,36,37,39–41], one in Romania [35], and one in South Korea [38]. Seven studies [16,33,34,36,37,39,41] evaluated the association between preoperative NLR and pre-operative DVT, while three [35,38,40] assessed the association between preoperative NLR and post-operative DVT. The details regarding the time of NLR measurement are provided in S3 Table. The surgical procedures involved were diverse and included patellar fractures, ankle fractures, tibial plateau fractures, femoral neck fractures, hip fractures, and total knee or joint arthroplasty. DVT was diagnosed using ultrasound in seven studies [16,33–36,39,41], CT in one study [38], and venography in one study [40]. However, this diagnostic method was not reported in one study [37]. The incidence of DVT ranges from 6.85% to 38.6%. Cut-off values for elevated NLR also varied considerably across studies, from 1.9 to 5.32. The study quality, assessed using NOS, was generally good, with scores ranging from 7 to 8 out of a possible 9 points (Tables 2 and S4).

**Table 2:** Characteristics of studies.

Studies	Age (years)	Male (%)	*n*	Procedures	Event time^†^	Incidence (%)	Cut-off	Diagnosis of DVT	Country	NOS
Diao 2022	57	61	500	Patella fractures	pre	7.8	4.6	Ultrasound	China	8
Gao 2023	42.8	59.6	1103	Ankle fracture	pre	8.3	4	Ultrasound	China	7
Liu 2020	45.6	62.9	1179	Tibial plateau fractures	pre	16.3	2.9	Ultrasound	China	8
Melinte 2022	66.4	44.7	273	TKR	post	10.3	3.88	Ultrasound	Romania	7
Niu 2022	71.7	29.9	708	Femoral neck fractures	pre	15.8	Q4 vs. Q1	Ultrasound	China	8
Peng 2021^⁋^	74-76	44.2	104	Hip fracture	pre	Na	na^§^	na	China	7
Seo 2021	70	10	264	TKR	post	38.6	1.9	CT	Korea	7
Xiong 2023	69.4	20.2	584	Total knee arthroplasty	pre	6.85	na	Ultrasound	China	8
Yao 2018	64.1	29	773	Total joint arthroplasty	post	15.5	na^§^	Venography	China	7
Zeng 2023	83	25.9	209	Femoral fractures	pre	19	5.32	Ultrasound	China	8

^⁋^Case-control study.

^§^Multiple logistic regression analyses; CT: venography computed tomography; NOS: Newcastle-Ottawa Scale scores.

^†^The time frame during which venous thrombosis occurred; pre: preoperative; post: postoperative; na: not available; TKR: total knee replacement.

### 3.3. Outcomes

#### 3.3.1. Incidence of DVT and association between NLR and DVT.

**Fig 2:**
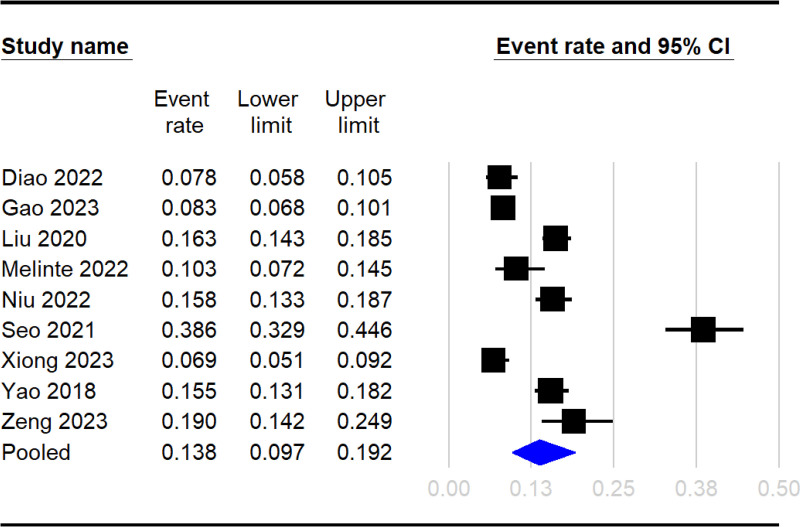
Pooled incidence of deep vein thrombosis (DVT) across the included studies. The forest plot shows individual study event rates (black squares) with 95% confidence intervals (horizontal lines) and the pooled estimate (blue diamond). The overall pooled DVT incidence was 13.8% (95% CI: 9.7%–19.2%). CI, confidence interval.

**Fig 3:**
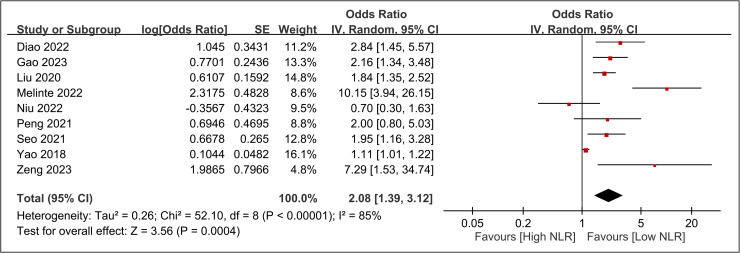
Forest plot demonstrating the association between elevated neutrophil-to-lymphocyte ratio (NLR) and risk of deep vein thrombosis (DVT). Odds ratios for individual studies are shown as squares with 95% confidence intervals (horizontal lines). The size of each square represents the weight of the study in the meta-analysis. Diamond represents the pooled odds ratio. CI, confidence interval.

**Fig 4:**
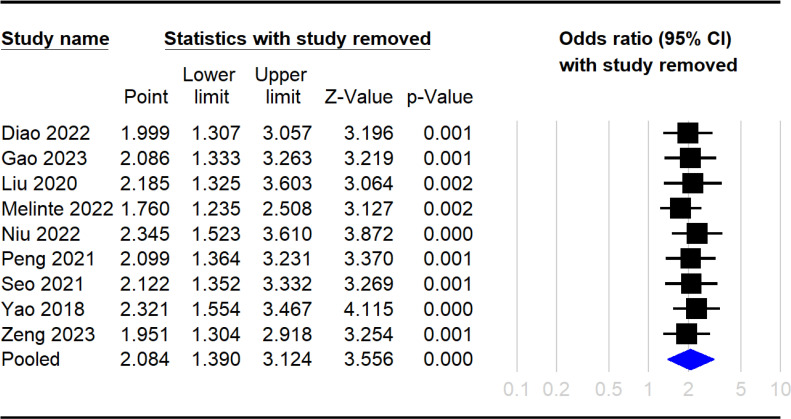
Leave-one-out sensitivity analysis showing the stability of the association between elevated NLR and DVT risk. Black squares represent the pooled odds ratio after removing each study with 95% confidence intervals (horizontal lines). The blue diamond shows the overall pooled estimate. The analysis demonstrated consistent statistical significance across all iterations. CI: confidence interval. An odds ratio >  1 indicates increased DVT risk with elevated NLR.

**Fig 5:**
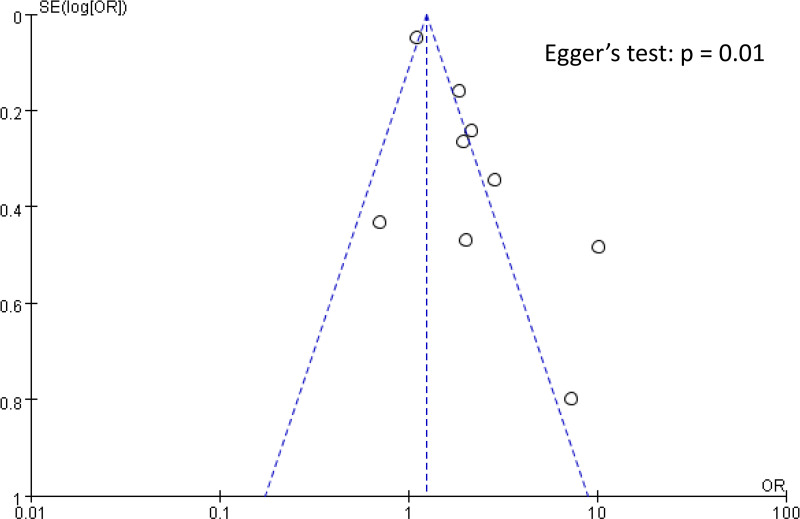
Funnel plot assessing publication bias for studies examining the association between elevated NLR and DVT risk. Each circle represents an individual study. The x-axis shows the odds ratio (OR) on a logarithmic scale, and the y-axis shows the standard error of the log [OR]. The dashed lines form a triangular region where 95% of the studies would be expected to lie in the absence of publication bias. Egger’s test (p =  0.01) indicates significant publication bias.

**Fig 6:**
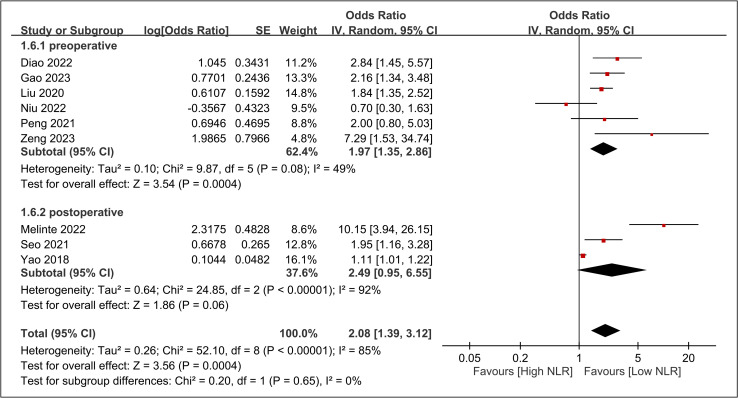
Subgroup analysis was performed based on the time frame during which deep vein thrombosis (DVT) occurred (preoperative vs. postoperative). CI, confidence interval.

The Raw data used in the current meta-analysis are available in S5 Table. The pooled incidence of DVT across the 10 included studies was 13.8% (95% CI: 9.7%–19.2%) (Fig 2) [16,33–41]. The pooled OR for the association between elevated NLR and DVT risk was 2.08 (95% CI: 1.39 to 3.12, p =  0.0004), indicating that patients with high NLR had approximately twice the odds of developing DVT compared to those with low NLR (Fig 3). In leave-one-out sensitivity analysis, the pooled OR remained statistically significant, ranging from 1.76 to 2.345 with the omission of any single study (Fig 4). However, there was significant heterogeneity across the studies (I^2^ =  85%, p <  0.00001). The funnel plot showed asymmetry (Fig 5), and Egger’s test indicated a significant risk of publication bias (p =  0.01). Subgroup analysis was performed based on the time frame during which DVT occurred (preoperative vs. postoperative) (Fig 6). The pooled OR was 1.97 (95% CI: 1.35 to 2.86, p = 0.0004, I^2^ = 49%) for studies focusing on the preoperative period and 2.49 (95% CI: 0.95 to 6.55, p = 0.06, I^2^ = 92%) for studies focusing on the postoperative period. The difference between subgroups was not statistically significant (p =  0.65 for subgroup difference) (Fig 6).

**Fig 7:**
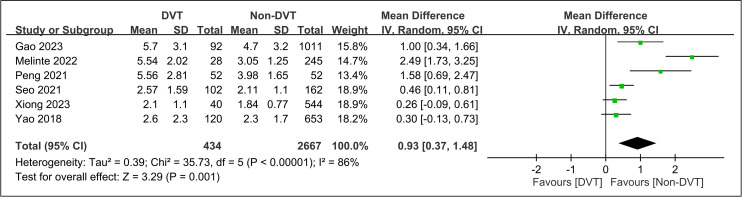
Forest plot showing the pooled mean difference in neutrophil-to-lymphocyte ratio (NLR) between patients who developed deep vein thrombosis (DVT) and those who did not. CI, confidence interval.

Across the six studies [34,35,37–40] that reported NLR as a continuous variable, the pooled mean difference in NLR between patients who developed DVT versus those who did not was 0.93 (95% CI: 0.37 to 1.48, p =  0.001, sensitivity analysis: consistent) (Fig 7). This indicates that, on average, patients with DVT had an NLR that was 0.93 units higher than that of patients without DVT. However, there was considerable heterogeneity between the studies (I^2^ =  86%, p <  0.00001).

#### 3.3.2. The association of patient characteristics with risk of DVT.

**Fig 8:**
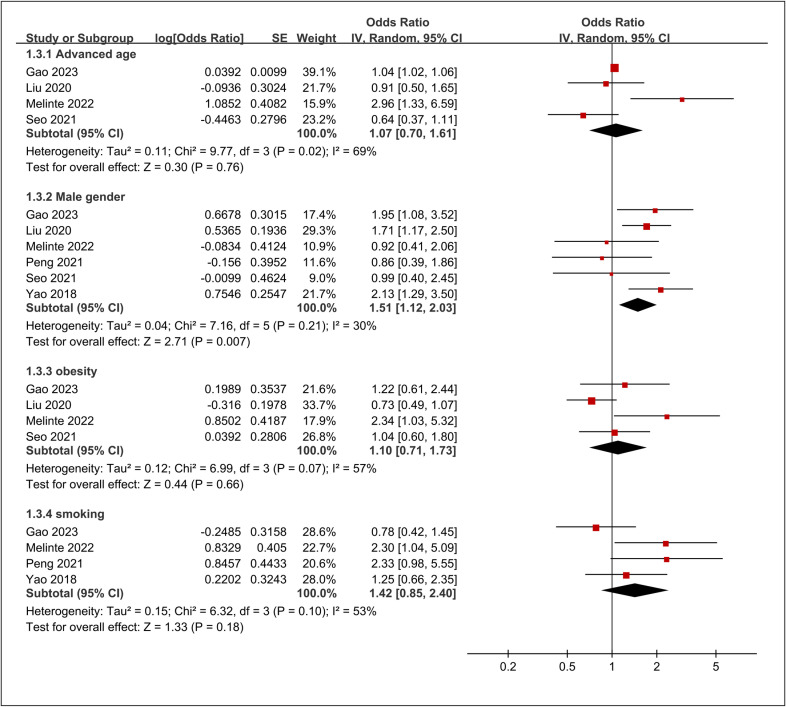
Forest plot showing the association between patient characteristics and risk of deep vein thrombosis (DVT). CI, confidence interval.

Four studies reported data on the association between advanced age (variably defined across studies) and DVT risk, with a pooled OR of 1.07 (95% CI: 0.70 to 1.61, p =  0.76, I^2^ = 69%) (Fig 8) [16,34,35,38]. Six studies provided data on the association between male sex and DVT risk [16,34,35,37,38,40] (Fig 8). The pooled OR was 1.51 (95% CI: 1.12 to 2.03, p =  0.007, I^2^ = 30%), suggesting a significantly increased risk of DVT in males compared to females. Four studies assessed the association between obesity and DVT risk, with a pooled OR of 1.10 (95% CI: 0.71 to 1.73, p =  0.66, I^2^ = 57%) (Fig 8) [16,34,35,38]. Four studies [34,35,37,40] evaluated the association between smoking and DVT risk (Fig 8). The pooled OR was 1.42 (95% CI: 0.85 to 2.40, p =  0.18, I^2^ = 53%), suggesting a non-significant trend towards increased risk in smokers. The results of the sensitivity analysis of these outcomes are summarized in Table 3.

**Table 3:** Summary of outcomes and certainty of evidence based on the grading of recommendations assessment, development and evaluation (GRADE) approach.

Outcomes	n	Certainty assessment (Domains)					Effect size [95% CI]	I^2^	Sensitivity analysis	Certainty
		A	B	C	D	E				
Elevated NLR (category variable) and DVT risk	9	NS	S	NS	NS	S	OR 2.08 [1.39, 3.12]	85%	Consistent	⨁◯◯◯ Very Low
Elevated NLR (continuous variable) and DVT risk^†^	6	NS	S	NS	NS	–	MD 0.93 [0.37, 1.48]	86%	Consistent	⨁◯◯◯ Very Low
Advanced age and DVT risk^†^	4	NS	NS	NS	NS	–	OR 1.07 [0.70, 1.61]	69%	Consistent	⨁⨁◯◯ Low
Male sex and DVT risk^†^	6	NS	NS	NS	NS	–	OR 1.51 [1.12, 2.03]	30%	Inconsistent	⨁⨁◯◯ Low
Obesity and DVT risk^†^	4	NS	NS	NS	NS	–	OR 1.10 [0.71, 1.73]	57%	Consistent	⨁⨁◯◯ Low
Smoking and DVT risk^†^	4	NS	NS	NS	NS	–	OR 1.42 [0.85, 2.40]	53%	Inconsistent	⨁⨁◯◯ Low
Cardiovascular disease and the DVT risk^†^	3	NS	NS	NS	NS	–	OR 1.32 [0.82 to 2.13]	0%	Consistent	⨁⨁◯◯ Low
Diabetes mellitus and DVT risk^†^	3	NS	NS	NS	NS	–	OR 1.60 [1.06 to 2.41]	0%	Inconsistent	⨁⨁◯◯ Low
Hypertension and DVT risk^†^	3	NS	NS	NS	NS	–	OR 1.43 [1.06 to 1.93]	0%	Inconsistent	⨁⨁◯◯ Low

A: risk of bias; B: Inconsistency; C: Indirectness; D: Imprecision; E: publication bias; N: not serious; S: serious.

^†^Publication bias was not assessed due to the limited number of available studies.

OR, odds ratio; MD, mean difference; CI, confidence interval; NLR, neutrophil-to-lymphocyte ratio; DVT, deep vein thrombosis.

In summary, male sex emerged as the only characteristic significantly associated with increased DVT risk, with 51% higher odds compared to females. Old age, obesity, and smoking showed non-significant associations with DVT risk, although smoking displayed a trend towards increased risk that did not reach statistical significance.

#### 3.3.3. The association of comorbidities with risk of DVT.

**Fig 9:**
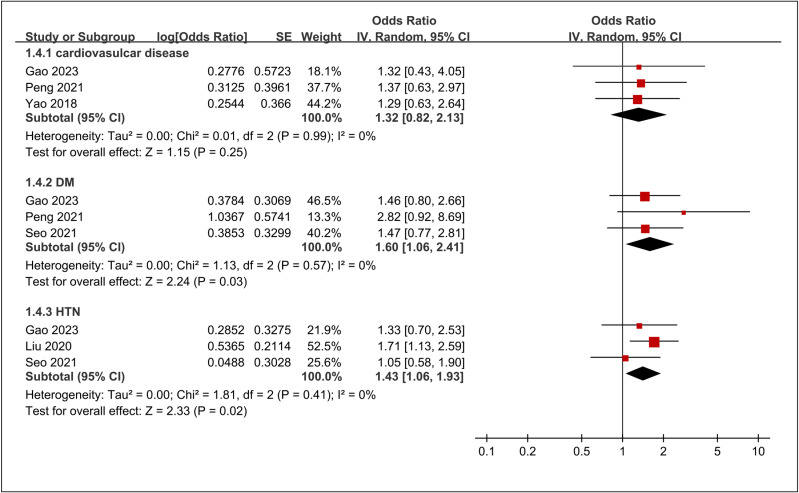
Forest plot showing the association between comorbidities and risk of deep vein thrombosis (DVT). CI, confidence interval; HTN, hypertension; DM, diabetes mellitus.

Three studies [34,37,40] examined the link between cardiovascular disease and the risk of DVT, yielding a combined OR of 1.32 (95% CI: 0.82 to 2.13, p =  0.25, I^2^ = 0%) (Fig 9). Similarly, three studies [34,37,38] explored the connection between diabetes mellitus and DVT risk, resulting in a pooled OR of 1.60 (95% CI: 1.06 to 2.41, p =  0.03, I^2^ = 0%), showing a statistically significant 60% increase in DVT risk among patients with diabetes (Fig 9). Another three studies [16,34,38] investigated the association between hypertension and DVT risk, with a pooled OR of 1.43 (95% CI: 1.06 to 1.93, p =  0.02, I^2^ = 0%), also indicating a significant association (Fig 9). Among the comorbidities studied, diabetes mellitus and hypertension were significantly associated with an increased risk of DVT, whereas cardiovascular disease did not demonstrate a significant impact on DVT risk. The results of the sensitivity analysis of these outcomes are summarized in Table 3.

### 3.4. Meta-regression analysis

**Fig 10:**
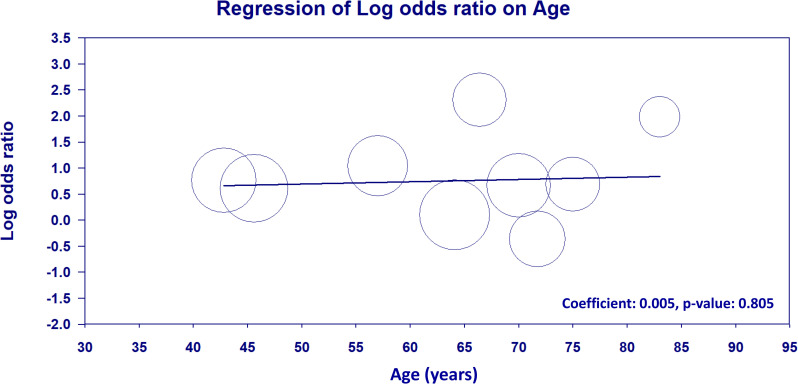
Meta-regression analysis of the association between age and log odds ratio of neutrophil-to-lymphocyte ratio (NLR) for deep vein thrombosis (DVT) risk. Each circle represents an individual study, with the circle size proportional to the study’s weight in the meta-regression analysis. The solid line represents the meta-regression line. The meta-regression analysis showed no significant association between age and the predictive value of NLR for DVT risk (coefficient: 0.005, p =  0.805), suggesting that the association between NLR and DVT risk is likely independent of age.

A meta-regression analysis was conducted to evaluate the potential effect of age on the relationship between NLR and DVT risk. The results showed no significant association between age and log OR of NLR and DVT risk (coefficient: 0.005, p =  0.805), indicating that the predictive value of NLR for DVT risk is likely independent of age (Fig 10).

### 3.5. Certainty of evidence

The certainty of evidence for the association between elevated NLR and DVT risk was assessed using the GRADE approach (Table 3). The certainty of evidence was rated very low for the association between an elevated NLR (both as a category variable and a continuous variable) and the risk of DVT. For the association between advanced age, male sex, obesity, smoking, cardiovascular disease, diabetes mellitus, and hypertension and the risk of DVT, the certainty of evidence was rated as low.

## 4. Discussion

This meta-analysis, encompassing 10 studies and 5,697 patients undergoing lower extremity orthopedic surgery, revealed several significant findings. The pooled incidence of DVT was 13.8%, underscoring its prevalence in this population. Notably, elevated NLR was associated with a two-fold increase in DVT risk (OR 2.08), with patients who developed DVT having, on average, a 0.93 unit higher NLR. Among the patient characteristics, male sex emerged as a significant risk factor (OR 1.51), while age, obesity, and smoking showed non-significant associations. Comorbidities such as diabetes mellitus (OR 1.60) and hypertension (OR 1.43) were significantly linked to increased DVT risk. Interestingly, subgroup analysis revealed no significant difference in NLR’s association with DVT risk between the pre- and postoperative periods. These findings collectively highlight the potential utility of NLR as a predictive marker for DVT risk in patients undergoing lower extremity orthopedic surgery and identify key risk factors for targeted prevention strategies.

The pooled incidence of DVT in patients undergoing lower extremity orthopedic surgery, as revealed by our meta-analysis, was 13.8% (95% CI: 9.7%–19.2%). This relatively high incidence highlights the critical need for effective risk assessment and prophylactic measures in orthopedic surgical practice. Notably, the incidence rates varied considerably across individual studies, ranging from 6.9% to 38.6%. This variability may be attributed to several factors, including differences in patient demographics, specific surgical procedures, prophylaxis protocols, and diagnostic methods. For instance, studies using more sensitive diagnostic techniques or those focusing on high-risk patient subgroups may report higher incidence rates.

Our meta-analysis revealed a significant association between an elevated NLR and an increased risk of DVT in patients undergoing lower extremity orthopedic surgery. This finding aligns with that of a previous meta-analysis [32], suggesting that NLR is a useful marker of thrombosis. Our meta-analysis has several significant strengths compared to the study by Hu et al. [32]. One of the primary advantages is the inclusion of 10 studies that specifically addressed lower extremity orthopedic surgery, whereas Hu et al. [32] only considered three such studies. By focusing on this homogeneous patient population, our analysis allows for more accurate and clinically relevant conclusions tailored to this group. Moreover, while Hu et al. [32] explored the diagnostic performance of NLR for DVT in a broader context, our study delves deeper into the specific relationship between NLR and the risk of DVT in patients undergoing lower extremity orthopedic surgery. This narrower focus provides more detailed and applicable insights for clinicians managing patients in this surgical category.

Our GRADE assessment rated the certainty of evidence for the primary outcome as “very low.” This rating indicates substantial uncertainty regarding the true effect size and suggests that the actual association may differ significantly from our meta-analysis estimates. Although our findings support the potential role of NLR in DVT risk assessment, clinicians should exercise caution when using NLR as a standalone predictor. We recommend that NLR be considered as part of a comprehensive risk assessment strategy rather than as an independent decision-making tool until higher-quality evidence becomes available.

It is important to acknowledge that NLR, as a marker of systemic inflammation, lacks specificity for thrombosis. An elevated NLR is associated with various conditions characterized by inflammation, such as infections, autoimmune diseases, and cancer. Therefore, the use of NLR as a standalone marker for thrombosis may be limited by the potential for false positives in non-thrombotic conditions. For example, COVID-19 is associated with a profound inflammatory response, and an elevated NLR may reflect the overall severity of the disease rather than a specific risk for thrombosis. It is crucial to consider the clinical context and the presence of other risk factors for thrombosis when interpreting the NLR results.

While our meta-analysis demonstrated an association between an elevated NLR and the risk of DVT, the significant heterogeneity observed across studies suggests that NLR alone may have limited predictive value. Combining NLR with other coagulation markers could potentially enhance its predictive performance for DVT in this patient population. D-dimer, a degradation product of cross-linked fibrin, is a well-established marker of coagulation activation and has been widely used for the diagnosis and risk stratification of DVT [42–45]. Elevated D-dimer levels have been consistently associated with an increased risk of DVT in various patient populations, including those undergoing orthopedic surgery [46–48]. A previous study showed that both NLR (OR: 2.636) and D-dimer levels (OR: 4.175) were associated with DVT in patients with unilateral limb edema [49]. While the effect size of NLR appears to be somewhat smaller than that reported for D-dimer, one potential advantage of NLR over D-dimer is its greater specificity for inflammation. D-dimer levels can be influenced by various factors, including age, pregnancy, malignancy, and inflammation, which may limit their specificity for DVT. In contrast, NLR is a more direct marker of systemic inflammation and plays a key role in the pathogenesis of DVT [50]. Combining NLR with D-dimer measurements may potentially enhance the predictive accuracy for DVT by capturing both the inflammatory and coagulation aspects of thrombosis pathophysiology [34]. Studies evaluating the incremental value of adding NLR to D-dimer-based risk assessment models in the setting of lower extremity orthopedic surgery are warranted.

The findings of this meta-analysis have important implications for clinical practice, policy development, and future research. Clinically, the strong link between elevated NLR and increased DVT risk suggests that NLR can be integrated into preoperative risk assessment protocols for patients undergoing lower extremity orthopedic surgery. To enhance clinical applicability of NLR, we propose integrating it with a threshold of 3.88 into existing DVT risk assessment protocols. This cutoff, supported by Melinte et al.’s study [35] showing excellent discriminative ability (AUC = 0.862), could supplement the established tools. This cost-effective biomarker has the potential to enhance current risk stratification strategies, leading to more personalized thromboprophylaxis measures and improved patient outcomes. High-risk patients identified by an elevated NLR may benefit from more aggressive preventive strategies or closer postoperative monitoring. From a policy standpoint, these results could inform the update of clinical guidelines for DVT prevention in orthopedic surgery, possibly leading to the routine inclusion of NLR testing in preoperative assessments. This could optimize resource allocation and improve patient care. Future studies should investigate the potential mechanisms underlying the relationship between elevated NLR and increased DVT risk. This could include exploring the roles of the systemic inflammation, immune response, and coagulation pathways. Methodological refinements, such as improved standardization of NLR measurement timing, more homogeneous patient populations, and adjustments for confounding variables, could further validate our findings and address the limitations of this study.

Our meta-analysis aimed to assess the association between elevated NLR and DVT risk in patients undergoing lower extremity orthopedic surgery. However, the findings should be interpreted with caution because of potential biases in the included studies. Inconsistency was observed for the primary outcome, as indicated by the high heterogeneity (I² >  75%) in the effect estimates. This inconsistency may be attributed to differences in study populations, NLR cutoff values, and diagnostic criteria for DVT across studies. Indirectness was not a major concern, as the included studies directly addressed the review question and the population of interest. Similarly, imprecision was not a concern due to the relatively narrow confidence interval (95% CI: 1.39, 3.12), which suggests a consistent effect size. Selection bias and measurement bias cannot be ruled out, as the majority of the included studies were retrospective observational studies with potential differences in patient characteristics and methods for diagnosing DVT. Finally, confounding factors such as disease severity and treatment strategies may have influenced the observed associations, and the extent to which these factors were accounted for varied across studies.

Several limitations of this meta-analysis should be considered when interpreting the results. First, the included studies were predominantly observational, which may have introduced inherent biases and limited causal inferences. Second, significant heterogeneity was observed across studies, potentially due to variations in patient populations, surgical procedures, NLR cutoff values, and DVT diagnostic criteria. This heterogeneity may have affected the generalizability of our findings. Third, most of the included studies were conducted in Asian populations, which may have limited the applicability of the results to other ethnicities and healthcare systems. For example, differences in healthcare delivery systems (e.g., prophylactic protocols, surgical techniques, and diagnostic approaches) across regions may affect both NLR measurements and DVT detection. Fourth, most of the included studies did not provide detailed information on the timing of NLR measurement (S3 Table), which unfortunately limited our ability to conduct a more in-depth analysis. Additionally, potential confounding factors, such as medication use (e.g., anticoagulants) and other inflammatory conditions, were not consistently reported or adjusted for in all studies. Fifth, another limitation was the relatively small number of included studies (n = 10), which limited both the precision of the pooled estimates and our ability to conduct detailed subgroup analyses to investigate heterogeneity. In particular, our meta-regression analysis only explored age as a potential effect modifier of the NLR-DVT relationship, while other important variables, such as sex, comorbidities, and surgical procedure types, could not be analyzed because of limited data availability and heterogeneous reporting across studies. Finally, there was evidence of publication bias, suggesting that studies with negative findings may be underrepresented in the published literature. To address this limitation, future systematic reviews should include unpublished data, conference proceedings, and dissertations through comprehensive searches of gray literature databases.

## 5. Conclusion

Our results indicate that patients with an elevated NLR have approximately double the odds of developing DVT than those with a lower NLR. This finding highlights the potential of NLR as an accessible and cost-effective biomarker for DVT risk assessment. Although NLR has potential as a risk stratification marker, its limitations should be recognized, and its value should be evaluated in conjunction with other biomarkers and clinical factors. Future studies should focus on establishing optimal NLR cutoff values, integrating NLR into existing risk assessment models, validating NLR-guided thromboprophylaxis strategies, and evaluating the cost-effectiveness of incorporating NLR assessments into routine preoperative care.

## Supporting information

S1 Table PRISMA checklist. (DOCX)

S2 Table Studies included and excluded. (DOCX)

S3 Table Details regarding preoperative neutrophile-to-lymphocyte ratio (NLR) measurement. (DOCX)

S4 Table Quality of studies based on Newcastle-Ottawa Scale scores. (DOCX)

S5 Table Raw data used in current meta-analysis. (DOCX)
