# Supplementary material for: Association between neutrophile-to-lymphocyte ratio and risk of deep vein thrombosis in patient receiving lower extremity orthopedic surgery: A meta-analysis
Source: PLoS One. 2025 Feb 24;20(2):e0319107. doi: 10.1371/journal.pone.0319107 (PMC11849845; doi:10.1371/journal.pone.0319107)
Supplement: S2 Table — (DOCX) [file pone.0319107.s002.docx]

**S2 Table.** Studies included and excluded

| **Studies identified during initial literature search (n=135) after remove duplicate studies** | |
| --- | --- |
| 1. Akcal, M. A., & Eke, I. (2021). Post-Operative Red Cell Distribution Width Increase May Predict Mortality in Patients Operated for Hip Fracture. *Clinical Laboratory, 67(11)*, 2568-2575. doi:https://dx.doi.org/10.7754/Clin.Lab.2021.210337 2. Akkaya, G., & Bilen, C. (2019). A Research for Predictive Value of Hemogram Parameters at Late Term Arteriovenous Fistula Thrombosis Formation. *E Journal of Cardiovascular Medicine, 7(1 Supplement)*, 69. 3. An, J., Han, L., Ma, X., Chang, Y., & Zhang, C. (2024). Influence of diabetes on the risk of deep vein thrombosis of patients after total knee arthroplasty: a meta-analysis. *Journal of Orthopaedic Surgery and Research, 19*(1), 164. 4. Anonymous. (2015). Poster Liver. *Journal of Gastroenterology and Hepatology (Australia), 30(Supplement 4)*, 323-407. doi:https://dx.doi.org/10.1111/jgh.13189 5. Aras, S., Emektar, E., Corbacioglu, S. K., & Cevik, Y. (2022). Evaluation of D-dimer and fibrinogen levels in COVID-19 patients D-dimer and fibrinogen levels in COVID-19 patients. *Annals of Clinical and Analytical Medicine, 13(7)*, 797-801. doi:https://dx.doi.org/10.4328/ACAM.21126 6. Arbănași, E. M., & Russu, E. (2024). The Prognostic Role of Neutrophil-to-Lymphocyte Ratio, Monocyte-to-Lymphocyte Ratio, and Platelet-to-Lymphocyte Ratio in the Risk of Major Adverse Cardiovascular Events and Mortality in Patients with COVID-19: a State-of-the-Art Review. *Journal of Cardiovascular Emergencies, 10*(2), 61-70. 7. Arnold, N. R., Samuel, L. T., Karnuta, J. M., Acuna, A. J., & Kamath, A. F. (2022). The international normalised ratio predicts perioperative complications in revision total hip arthroplasty. *Hip International, 32(5)*, 661-671. doi:https://dx.doi.org/10.1177/1120700020973972 8. Barker, T., Rogers, V. E., Henriksen, V. T., Brown, K. B., Trawick, R. H., Momberger, N. G., & Lynn Rasmussen, G. (2016). Is there a link between the neutrophil-to-lymphocyte ratio and venous thromboembolic events after knee arthroplasty? A pilot study. *Journal of Orthopaedics and Traumatology, 17*(2), 163-168. doi:10.1007/s10195-015-0378-3 9. Bawa, H., Weick, J. W., Dirschl, D. R., & Luu, H. H. (2018). Trends in Deep Vein Thrombosis Prophylaxis and Deep Vein Thrombosis Rates After Total Hip and Knee Arthroplasty. *Journal of the American Academy of Orthopaedic Surgeons, 26*(19), 698-705. doi:10.5435/jaaos-d-17-00235 10. Bekki, A., Garcia-Ortega, A., Lopez-Reyes, R., Oscullo, G., Beauperthuy, T., Siddiqui, F., . . . Monreal, M. (2022). Cellular indices and outcome in patients with acute venous thromboembolism. *European Respiratory Journal. Conference: European Respiratory Society International Congress, ERS, 60*(Supplement 66). doi:https://dx.doi.org/10.1183/13993003.congress-2022.321 11. Beyazal, O. F. (2022). Does Complete Blood Count Have A Role In The Prediction Of Deep Vein Thrombosis? *E Journal of Cardiovascular Medicine. Conference: 18th International Congress of Update in Cardiology and Cardiovascular Surgery. Ankara Turkey, 10*(1). 12. Bhat, T. M., Afari, M. E., & Garcia, L. A. (2016). Neutrophil lymphocyte ratio in peripheral vascular disease: a review. *Expert Review of Cardiovascular Therapy, 14(7)*, 871-875. doi:https://dx.doi.org/10.1586/14779072.2016.1165091 13. Billoir, P., Siguret, V., Drouet, L., Masson Fron, E., Bagan Triquenot, A., Crassard, I., & Le Cam Duchez, V. (2022). Association of inflammation and coagulation biomarkers with cerebral venous thrombosis clinical and imaging characteristics: Results from FPCCVT. *Research and Practice in Thrombosis and Haemostasis. Conference, 6*(Supplement 1). doi:https://dx.doi.org/10.1002/rth2.12787 14. Calfon, M., Seddighzadeh, A., Piazza, G., & Goldhaber, S. Z. (2009). Deep vein thrombosis in orthopedic surgery. *Clinical and Applied Thrombosis/Hemostasis, 15*(5), 512-516. 15. Candreva, A., & Matter, C. M. (2022). Is the amount of glow predicting the fire? Residual inflammatory risk after percutaneous coronary intervention. *European Heart Journal, 43(7)*, E10-E13. doi:https://dx.doi.org/10.1093/eurheartj/ehy729 16. Cavalca, F., Palandri, F., Pugliese, N., Ripamonti, A., Di Veroli, A., Biondo, M., . . . Elli, E. M. (2022). Neutrophil to Lymphocyte Ratio (NLR) at Diagnosis Is a Predictor of Venous Thrombosis and Poor Outcome in Patients with Prefibrotic Primary Myelofibrosis. *Blood, 140(Supplement 1)*, 9711-9712. doi:https://dx.doi.org/10.1182/blood-2022-165456 17. Chacko, B., Thomas, L., Sharma, R., Yadav, B., Jeyaseelan, L., Arul, A. O., . . . Peter, J. V. (2022). Noninvasive Ventilation in the Management of Respiratory Failure Due to COVID-19 Infection: Experience From a Resource-Limited Setting. *Mayo Clinic Proceedings, 97(1)*, 31-45. doi:https://dx.doi.org/10.1016/j.mayocp.2021.10.002 18. Chang, W., Wang, B., Li, Q., Zhang, Y., & Xie, W. (2021). Study on the risk factors of preoperative deep vein thrombosis (DVT) in patients with lower extremity fracture. *Clinical and Applied Thrombosis/Hemostasis, 27*, 10760296211002900. 19. Chen, G., Xie, X., Wang, M., Guo, X., Zhang, Z., Zhang, L., & Zhang, B. (2022). Prognostic Significance of Tumor Growth Rate (TGR) in Patients with Huge Hepatocellular Carcinoma Undergoing Transcatheter Arterial Chemoembolization. *Current Oncology, 29(2)*, 423-432. doi:https://dx.doi.org/10.3390/curroncol29020038 20. Chen, W., Li, Y., Guo, D., & Xia, S. (2022). Evaluation and Clinical Value of Lipid Metabolites in the Prognosis of Acute Pulmonary Thromboembolism. *Pulmonary Circulation. Conference, 12*(4). doi:https://dx.doi.org/10.1002/pul2.12153 21. Chen, X., Fan, Y., Tu, H., Chen, J., & Li, R. (2024). A nomogram model based on the systemic immune-inflammation index to predict the risk of venous thromboembolism in elderly patients after hip fracture: A retrospective cohort study. *Heliyon, 10*(6). 22. Chen, Z. H., Lin, J. X., Hong, Y. F., Dong, M., Lin, Q., & Wu, X. Y. (2016). Predictive value of neutrophil-to-lymphocyte ratio (NLR), plateletto- lymphocyte ratio (PLR), and lymphocyte-to-monocyte ratio (LMR) for patients with advanced HBV-associated HCC. *Journal of Clinical Oncology. Conference, 34*(Supplement 15). doi:https://dx.doi.org/10.1200/JCO.2016.34.15-suppl.e15609 23. Chenevier-Gobeaux, C., Ducastel, M., Meritet, J. F., Ballaa, Y., Chapuis, N., Pene, F., . . . Borderie, D. (2022). Plasma Endocan as a Biomarker of Thrombotic Events in COVID-19 Patients. *Journal of Clinical Medicine, 11(19) (no pagination)*(5560). doi:https://dx.doi.org/10.3390/jcm11195560 24. Cheng, J., Fu, Z., Zhu, J., Zhou, L., & Song, W. (2020). The predictive value of plasminogen activator inhibitor-1, fibrinogen, and D-dimer for deep venous thrombosis following surgery for traumatic lower limb fracture. *Annals of palliative medicine, 9*(5), 3385392-3383392. 25. Chenming Hu, B., Bin Zhao, B., Qianling Ye, B., Jun Zou, B., & Xiang Li, M. (2023). The Diagnostic Value of the Neutrophil-to-Lymphocyte Ratio and Platelet-to-Lymphocyte Ratio for Deep Venous Thrombosis: A Systematic Review and Meta-Analysis. 26. Cho, E. S., McClelland, P. H., Cheng, O., Kim, Y., Hu, J., Zenilman, M. E., & D'Ayala, M. (2021). Utility of d-dimer for diagnosis of deep vein thrombosis in coronavirus disease-19 infection. *Journal of Vascular Surgery: Venous and Lymphatic Disorders, 9*(1), 47-53. 27. Choi, M. C., Min, E. K., Lee, J. G., Joo, D. J., Kim, M. S., & Kim, D. G. (2022). Antiplatelet Drugs on the Recurrence of Hepatocellular Carcinoma after Liver Transplantation. *Cancers, 14(21) (no pagination)*(5329). doi:https://dx.doi.org/10.3390/cancers14215329 28. Coremans, L., Strubbe, B., & Peeters, H. (2021). Venous thromboembolism in patients with inflammatory bowel disease: review of literature and practical algorithms. *Acta Gastroenterologica Belgica, 84*(1), 79-85. doi:10.51821/84.1.910 29. Coskun, B., Ayhan, M., & Ulusoy, S. (2024). Relationship between Prognostic Nutritional Index and Amputation in Patients with Diabetic Foot Ulcer. *Diagnostics, 14(7) (no pagination)*(738). doi:https://dx.doi.org/10.3390/diagnostics14070738 30. Davidovic, A., Cvijanovic, D., Davidovic, J., Lazic, S., Lazic, B., Cucic, L., . . . Nikolic, N. M. (2022). GRACE, SYNTAX I, and SYNTAX II scores as predictors of one-year MACE in patients with myocardial infarction treated with percutaneous coronary intervention. *Vojnosanitetski Pregled, 79(9)*, 868-877. doi:https://dx.doi.org/10.2298/VSP200204055D 31. Debora, L., Suprapti, B., Kusumawati, D., Arina Dery, P. S., Gabriella Nathasya, T., Arini, M. N., & Aryanti, L. D. (2022). Analysis of Enoxaparin Effectiveness Based on COVID-19 Severity: A Study in a Secondary Hospital in Bandung, Indonesia. *Indonesian Journal of Pharmacy, 33(3)*, 381-393. doi:https://dx.doi.org/10.22146/ijp.4133 32. Demirkiran, N. D., & Ozmanevra, R. (2020). Neutrophil to lymphocyte ratio of patients who underwent bilateral versus unilateral unicompartmental knee arthroplasty. *Medicine, 9*(1), 227-230. 33. Demiroluk, O., Yigit, Y., Abitagaoglu, S., Ari, Z. B., & Arzu Yildirim, A. (2022). The predictive role of Troponin I levels for mortality in geriatric patients transferred to the intensive care unit for COVID-19 pneumonia. *Annals of Clinical and Analytical Medicine, 13(2)*, 200-205. doi:https://dx.doi.org/10.4328/ACAM.20907 34. Desilets, A., Harnois, M., Busque, L., & Szuber, N. (2022). Prognostic Impact of High Calreticulin Variant Allele Frequency in Patients with Myeloproliferative Neoplasms. *Blood, 140(Supplement 1)*, 9700-9701. doi:https://dx.doi.org/10.1182/blood-2022-163785 35. Diao, S., Li, J., Zhao, J., Wang, D., Wang, H., Xu, X., & Zhou, J. (2022). Risk factors and new inflammatory indicators of deep vein thrombosis after adult patella fractures. *Frontiers in surgery, 9*, 1028542. 36. Dikmen, Z. G. (2022). Classic and New Biomarkers of Inflammation. *Turkish Journal of Biochemistry, 47(Supplement 1)*, 14-15. 37. Ding, J., Yue, X., Tian, X., Liao, Z., Meng, R., & Zou, M. (2023). Association between inflammatory biomarkers and venous thromboembolism: a systematic review and meta-analysis. *Thromb J, 21*(1), 82. doi:10.1186/s12959-023-00526-y 38. Donazzan, L., Ruzzarin, A., Zilio, F., Muraglia, S., Zanon, S., Flaim, M., . . . Unterhuber, M. (2022). Usefulness of Neutrophil-to-Limphocyte Ratio as a Prognostic Biomarker for in-Hospital Complications and Long-Term Mortality in Oldest Old Stemi Patients. *European Heart Journal, Supplement, 24(Supplement K)*, K133. doi:https://dx.doi.org/10.1093/eurheartjsupp/suac121.370 39. Dou, J. P., Yu, J., Yang, X. H., Cheng, Z. G., Han, Z. Y., Liu, F. Y., . . . Liang, P. (2017). Outcomes of microwave ablation for hepatocellular carcinoma adjacent to large vessels: A propensity score analysis. *Oncotarget, 8(17)*, 28758-28768. doi:https://dx.doi.org/10.18632/oncotarget.15672 40. Ebner, M., Sentler, C., Harjola, V. P., Bueno, H., Lerchbaumer, M. H., Hasenfuss, G., . . . Lankeit, M. (2021). Outcome of patients with different clinical presentations of high-risk pulmonary embolism. *European Heart Journal: Acute Cardiovascular Care, 10(7)*, 787-796. doi:https://dx.doi.org/10.1093/ehjacc/zuab038 41. Esmon, C. T., Xu, J., & Lupu, F. (2011). Innate immunity and coagulation. *Journal of Thrombosis and Haemostasis, 9 Suppl 1*(Suppl 1), 182-188. doi:10.1111/j.1538-7836.2011.04323.x 42. Esposito, P., Picciotto, D., Cappadona, F., Russo, E., Falqui, V., Conti, N. E., . . . Viazzi, F. (2022). The Evolving Scenario of COVID-19 in Hemodialysis Patients. *International Journal of Environmental Research and Public Health, 19(17) (no pagination)*(10836). doi:https://dx.doi.org/10.3390/ijerph191710836 43. Falcinelli, E., Petito, E., & Gresele, P. (2022). The role of platelets, neutrophils and endothelium in COVID-19 infection. *Expert Review of Hematology, 15(8)*, 727-745. doi:https://dx.doi.org/10.1080/17474086.2022.2110061 44. Feng, L., Xie, Z., Zhou, X., Hou, C., Liang, Z., Lu, H., . . . Zhang, D. (2023). Diagnostic value of D-dimer for lower extremity deep venous thrombosis caused by rib fracture: a retrospective study. *Journal of Orthopaedic Surgery and Research, 18*(1), 515. 45. Ferroni, P., Riondino, S., Formica, V., Cereda, V., Tosetto, L., La Farina, F., . . . Roselli, M. (2015). Venous thromboembolism risk prediction in ambulatory cancer patients: clinical significance of neutrophil/lymphocyte ratio and platelet/lymphocyte ratio. *International Journal of Cancer, 136*(5), 1234-1240. doi:10.1002/ijc.29076 46. Forget, P., Moreau, N., Engel, H., Cornu, O., Boland, B., De Kock, M., & Yombi, J.-C. (2015). The neutrophil-to-lymphocyte ratio (NLR) after surgery for hip fracture (HF). *Archives of Gerontology and Geriatrics, 60*(2), 366-371. 47. Freund, Y., Cohen-Aubart, F., & Bloom, B. (2022). Acute pulmonary embolism: a review. *JAMA, 328*(13), 1336-1345. 48. Fu, Y.-H., Liu, P., Xu, X., Wang, P.-F., Shang, K., Ke, C., . . . Zhuang, Y. (2020). Deep vein thrombosis in the lower extremities after femoral neck fracture: a retrospective observational study. *Journal of Orthopaedic Surgery, 28*(1), 2309499019901172. 49. Gao, Z., Zhao, K., Jin, L., Lian, X., Zhang, Z., Ma, L., & Hou, Z. (2023). Combination of neutrophil to lymphocyte ratio, platelet to lymphocyte ratio with plasma D-dimer level to improve the diagnosis of deep venous thrombosis (DVT) following ankle fracture. *Journal of Orthopaedic Surgery and Research, 18*(1), 362. 50. Genchi, A., Semerano, A., Schwarz, G., Dell'Acqua, B., Gullotta, G. S., Sampaolo, M., . . . Bacigaluppi, M. (2022). Neutrophils predominate the immune signature of cerebral thrombi in COVID-19 stroke patients. *Acta neuropathologica communications, 10(1)*, 14. doi:https://dx.doi.org/10.1186/s40478-022-01313-y 51. Gharib, H. K., Szpunar, S., Bhargava, A., & Kafri, Z. (2023). Neutrophil-Lymphocyte Ratio and Platelet-Lymphocyte Ratio for an Early Detection of Venous Thromboembolism in COVID-19 Patients. *Blood, 142*, 5521. 52. Gharib, H. K., Szpunar, S., Bhargava, A., & Kafri, Z. (2023). Neutrophil-Lymphocyte Ratio and Platelet-Lymphocyte Ratio for an Early Detection of Venous Thromboembolism in COVID-19 Patients. *Blood, 142*, 5521. 53. Glavinic, R., Marcic, L., Dumancic, S., Pavicic Ivelja, M., Jelicic, I., Kalibovic Govorko, D., & Medvedec Mikic, I. (2022). Acute Arterial Thrombosis of Lower Extremities in COVID-19 Patients. *Journal of Clinical Medicine, 11(6) (no pagination)*(1538). doi:https://dx.doi.org/10.3390/jcm11061538 54. Grafeneder, J., Buchtele, N., Egger, D., Schwameis, M., Ay, C., Jilma, B., & Schoergenhofer, C. (2022). Disseminated Intravascular Coagulation Score Predicts Mortality in Patients with Liver Disease and Low Fibrinogen Level. *Thrombosis and Haemostasis, 122(12)*, 1980-1987. doi:https://dx.doi.org/10.1055/a-1925-2300 55. Gramaca, J., Gomes, S. G., Silva, M. F., Ponte, T., Carneiro, F., & Campante, F. (2019). Neutrophil-to-Lymphocyte Ratio and Platelet-to-Lymphocyte Ratio as Predictors of Venous Thromboembolism: A Retrospective, Single-Center Analysis. *European Journal of Case Reports in Internal Medicine, 6(Supplement 1)*, 134-135. doi:https://dx.doi.org/10.12890/2019_V6Sup1 56. Guzel, A., Gurmen, A., Tuysuz, S., Guclu, O., Huseyin, S., & Canbaz, S. (2021). Evaluation of the Efficiency of Novel Oral Anticoagulants and Warfarin in Deep Vein Thrombosis with Simple Blood Parameters. *E Journal of Cardiovascular Medicine. Conference: 17th International Congress of Update in Cardiology and Cardiovascular Surgery. Virtual., 9*(1). 57. He, M., Fan, Q., Zhu, Y., Liu, D., Liu, X., Xu, S., . . . Zhu, Z. (2021). The need for nutritional assessment and interventions based on the prognostic nutritional index for patients with femoral fractures: a retrospective study. *Perioperative Medicine, 10(1) (no pagination)*(61). doi:https://dx.doi.org/10.1186/s13741-021-00232-1 58. He, S.-y., Zhang, P., Qin, H.-j., Jiang, N., & Yu, B. (2022). Incidence and risk factors of preoperative deep venous thrombosis following hip fracture: a retrospective analysis of 293 consecutive patients. *European Journal of Trauma and Emergency Surgery, 48*(4), 3141-3147. 59. Hekimian, G., Masi, P., Lejeune, M., Lebreton, G., Chommeloux, J., Desnos, C., . . . Frere, C. (2021). Extracorporeal Membrane Oxygenation Induces Early Alterations in Coagulation and Fibrinolysis Profiles in COVID-19 Patients with Acute Respiratory Distress Syndrome. *Thrombosis and Haemostasis, 121(8)*, 1031-1042. doi:https://dx.doi.org/10.1055/a-1529-2257 60. Hernandez-Molina, G., Llorente-Chavez, A., & Martin-Nares, E. (2021). Thrombosis and Thrombocytopenia in Antiphospholipid Syndrome: Their Association with Mean Platelet Volume and Hematological Ratios. *European Journal of Case Reports in Internal Medicine, 8(Supplement 1)*, 373. doi:https://dx.doi.org/10.12890/2021_V8Sup1 61. Hillary, T., Clijmans, J., Vermeire, S., Lambert, J., Garmyn, M., Imbrechts, M., & Vanassche, T. (2021). Venous thrombotic events in psoriasis patients: a systematic review with meta-analysis. *Annals of Medicine, 53*(1), 1074-1081. doi:10.1080/07853890.2021.1942974 62. Hong, S. W., Jeong, H. C., & Kim, S. H. (2023). The Neutrophil-to-Lymphocyte Ratio and Preoperative Pulmonary Function Test Results as Predictors of In-Hospital Postoperative Complications after Hip Fracture Surgery in Older Adults +. *Journal of Clinical Medicine, 12(1) (no pagination)*(108). doi:https://dx.doi.org/10.3390/jcm12010108 63. Howell, J. A., Pinato, D. J., Ramaswami, R., Arizumi, T., Ferrari, C., Gibbin, A., . . . Sharma, R. (2017). Integration of the cancer-related inflammatory response as a stratifying biomarker of survival in hepatocellular carcinoma treated with sorafenib. *Oncotarget, 8(22)*, 36161-36170. doi:https://dx.doi.org/10.18632/oncotarget.15322 64. Hu, C., Zhao, B., Ye, Q., Zou, J., Li, X., & Wu, H. (2023). The Diagnostic Value of the Neutrophil-to-Lymphocyte Ratio and Platelet-to-Lymphocyte Ratio for Deep Venous Thrombosis: A Systematic Review and Meta-Analysis. *Clinical and Applied Thrombosis/Hemostasis, 29*, 10760296231187392. 65. Hu, Y., Zhu, L., Tian, X., & Duan, F. (2023). Prevalence of preoperative deep vein thrombosis in long bone fractures of lower limbs: a systematic review and meta-analysis. *Journal of Orthopaedics and Traumatology, 24*(1), 19. 66. Hu, Y., Zhu, L., Tian, X., & Duan, F. (2023). Prevalence of preoperative deep vein thrombosis in long bone fractures of lower limbs: a systematic review and meta-analysis. *Journal of Orthopaedics and Traumatology, 24*(1), 19. 67. Huang, L., Li, J., & Jiang, Y. (2016). Association between hypertension and deep vein thrombosis after orthopedic surgery: a meta-analysis. *European Journal of Medical Research, 21*, 1-7. 68. Jiang, S. Y., Huang, X. Q., Ni, L. Y., Wu, L., Ai, Y. J., & Chen, S. Y. (2022). Prognostic importance of interleukin 2 receptor for patients with a history of cirrhotic variceal bleeding. *Journal of Digestive Diseases, 23(10)*, 577-586. doi:https://dx.doi.org/10.1111/1751-2980.13141 69. Jin, W. Y., Pang, Y., Zhang, X. C., Peng, D. L., Yan, Z. W., Pan, S., . . . Zheng, X. Gender Differences do not Influence the Blood Coagulopathy in Patients Undergoing Total Knee Arthroplasty: A Retrospective Thromboelastography Analysis. *Clinical and Applied Thrombosis/Hemostasis, 27*, 10760296211055716. doi:https://dx.doi.org/10.1177/10760296211055716 70. Kanchanabat, B., Stapanavatr, W., Meknavin, S., Soorapanth, C., Sumanasrethakul, C., & Kanchanasuttirak, P. (2011). Systematic review and meta-analysis on the rate of postoperative venous thromboembolism in orthopaedic surgery in Asian patients without thromboprophylaxis. *Journal of British Surgery, 98*(10), 1356-1364. 71. Kantarcioglu, B., Pozin, J., O'Hara, A., Patil, R., Allen, M., Krupa, E., . . . Fareed, J. (2022). The Role of Blood Cellular Indices in Diabetic Pulmonary Embolism Patients in Predicting Adverse Outcomes. *Blood, 140(Supplement 1)*, 11228-11229. doi:https://dx.doi.org/10.1182/blood-2022-165765 72. Kekec, A. F., & Colak, T. S. (2022). Is there a predictive value of the preoperative neutrophil-lymphocyte ratio in terms of intensive care need in geriatric patients who underwent pertrochanteric fracture surgery? [Pertrokanterik femur kirigi geciren geriatrik hastalarda preoperatif notrofil-lenfosit oraninin yogun bakim ihtiyaci acisindan prediktif degeri var mi?.]. *Ulusal Travma ve Acil Cerrahi Dergisi, 28(8)*, 1164-1169. doi:https://dx.doi.org/10.14744/tjtes.2021.73404 73. Kobayashi, T., Akiyama, T., & Mawatari, M. (2023). Predictors of preoperative deep vein thrombosis in hip fractures: a systematic review and meta-analysis. *Journal of Orthopaedic Science, 28*(1), 222-232. 74. Kondo, Y., Kaneko, Y., Takei, H., Tamai, H., Kabata, H., Suhara, T., . . . Takeuchi, T. (2021). COVID-19 shares clinical features with anti-melanoma differentiation-associated protein 5 positive dermatomyositis and adult Still's disease. *Clinical and Experimental Rheumatology, 39(3)*, 631-638. doi:https://dx.doi.org/10.55563/CLINEXPRHEUMATOL/44KAJI 75. Kripalani, Y., & Parulekar, L. (2021). Pulmonary Embolism in a COVID-19-Positive Primigravida after Caesarean Section despite Prophylaxis. *European Journal of Case Reports in Internal Medicine, 8*(7). doi:https://dx.doi.org/10.12890/2021_002684 76. Kunutsor, S. K., Seidu, S., Blom, A. W., Khunti, K., & Laukkanen, J. A. (2017). Serum C-reactive protein increases the risk of venous thromboembolism: a prospective study and meta-analysis of published prospective evidence. *European Journal of Epidemiology, 32*(8), 657-667. doi:10.1007/s10654-017-0277-4 77. Kuplay, H., Erdoğan, S. B., Bastopcu, M., Arslanhan, G., Baykan, D. B., & Orhan, G. (2020). The neutrophil-lymphocyte ratio and the platelet-lymphocyte ratio correlate with thrombus burden in deep venous thrombosis. *Journal of Vascular Surgery: Venous and Lymphatic Disorders, 8*(3), 360-364. 78. Kyrle, P. A., & Eichinger, S. (2005). Deep vein thrombosis. *The Lancet, 365*(9465), 1163-1174. 79. Li, J., Zhu, Y., Chen, W., Zhao, K., Zhang, J., Meng, H., . . . Zhang, Y. (2020). Incidence and locations of deep venous thrombosis of the lower extremity following surgeries of tibial plateau fractures: a prospective cohort study. *Journal of Orthopaedic Surgery and Research, 15*(1), 605. doi:10.1186/s13018-020-02136-0 80. Li, Y., Jiang, Q., Zhou, X., Wu, M., Chen, J., Liu, H., . . . Zhao, S. (2022). A prospective marker for the prediction of postoperative deep venous thrombosis: Neutrophil extracellular traps. *Frontiers in Cell and Developmental Biology, 10(no pagination)*(1071550). doi:https://dx.doi.org/10.3389/fcell.2022.1071550 81. Lin, B., Lin, J., Wang, F., Wang, Y., Shen, S., Hong, X., . . . Yang, H. (2023). Computed tomography-defined sarcopenia as a risk factor for short-term postoperative complications in oral cancer patients with free flap reconstruction: A retrospective population-based cohort study. *Head and Neck, 45(10)*, 2555-2570. doi:https://dx.doi.org/10.1002/hed.27479 82. Linkins, L. A., & Takach Lapner, S. (2017). Review of D-dimer testing: Good, Bad, and Ugly. *International Journal of Laboratory Hematology, 39 Suppl 1*, 98-103. doi:10.1111/ijlh.12665 83. Liu, D., Zhu, Y., Chen, W., Li, J., Zhao, K., Zhang, J., . . . Zhang, Y. (2020). Relationship between the inflammation/immune indexes and deep venous thrombosis (DVT) incidence rate following tibial plateau fractures. *Journal of Orthopaedic Surgery and Research, 15*, 1-8. 84. Liu, D., Zhu, Y., Chen, W., Li, J., Zhao, K., Zhang, J., . . . Zhang, Y. (2020). Relationship between the inflammation/immune indexes and deep venous thrombosis (DVT) incidence rate following tibial plateau fractures. *Journal of Orthopaedic Surgery and Research, 15*, 1-8. 85. Liu, H., Chen, X., Wang, Z., Liu, Y., & Liu, M. (2023). High systemic inflammation response index level is associated with an increased risk of lower extremity deep venous thrombosis: a large retrospective study. *Annals of Medicine, 55*(2), 2249018. 86. Ma, J., Qin, J., Hu, J., Shang, M., Zhou, Y., Liang, N., . . . Zhu, Y. (2020). Incidence and hematological biomarkers associated with preoperative deep venous thrombosis following foot fractures. *Foot and Ankle International, 41*(12), 1563-1570. 87. Maggioni, A. P., Iervolino, A., & Andreotti, F. (2021). Is it time to introduce anti-inflammatory drugs into secondary cardiovascular prevention: Evidence from clinical trials? *Vessel Plus, 5(no pagination)*(14). doi:https://dx.doi.org/10.20517/2574-1209.2021.05 88. Melinte, R. M., Arbănași, E. M., Blesneac, A., Zolog, D. N., Kaller, R., Mureșan, A. V., . . . Russu, E. (2022). Inflammatory biomarkers as prognostic factors of acute deep vein thrombosis following the total knee arthroplasty. *Medicina, 58*(10), 1502. 89. Mohamed, I. H., Chowdary, P. B., Shetty, S., Sammartino, C., Sivaprakasam, R., Lindsey, B., . . . Khurram, M. A. (2021). Outcomes of Renal Transplant Recipients With SARS-CoV-2 Infection in the Eye of the Storm: A Comparative Study With Waitlisted Patients. *Transplantation, 105(1)*, 115-120. doi:https://dx.doi.org/10.1097/TP.0000000000003406 90. Momose, T., Nakano, M., Nakamura, Y., Maeda, T., & Nawata, M. (2024). Incidence and preventive treatment for deep vein thrombosis with our own preventive protocol in total hip and knee arthroplasty. *PloS One, 19*(1), e0293821. doi:10.1371/journal.pone.0293821 91. Nery, F., Carneiro, P., Correia, S., Macedo, C., Gandara, J., Lopes, V., . . . Valla, D. (2021). Systemic inflammation as a risk factor for portal vein thrombosis in cirrhosis: A prospective longitudinal study. *European Journal of Gastroenterology and Hepatology, 33(1 Supplement)*, E108-E113. doi:https://dx.doi.org/10.1097/MEG.0000000000001982 92. Nguyen, H. T., Vu, M. P., Nguyen, T. T. M., Nguyen, T. T., Kieu, T. V. O., Duong, H. Y., . . . Hoang, T. H. (2024). Association of the neutrophil-to-lymphocyte ratio with the occurrence of venous thromboembolism and arterial thrombosis. *Journal of International Medical Research, 52*(4), 03000605241240999. 93. Niu, S., Pei, Y., Hu, X., Ding, D., & Jiang, G. (2022). Relationship between the neutrophil-to-lymphocyte ratio or platelet-to-lymphocyte ratio and deep venous thrombosis (DVT) following femoral neck fractures in the elderly. *Frontiers in Surgery, 9*, 1001432. 94. Osunronbi, T., Olukoya, O., Jesuyajolu, D., Alare, K., Alemenzohu, H. O., Bello, R. O., . . . Sharma, H. (2024). The prognostic utility of neutrophil-lymphocyte ratio in spinal surgery: A systematic review and meta-analysis. *Journal of Clinical Neuroscience, 121*, 161-168. doi:https://dx.doi.org/10.1016/j.jocn.2024.02.021 95. Park, M. S., Seo, W. W., Kim, S. E., Lee, J. H., Park, D. G., Han, K. R., & Oh, D. J. (2017). Neutrophil lymphocyte ratio as a predictor of venous thromboembolism after total knee replacement. *European Heart Journal, 38(Supplement 1)*, 348-349. doi:https://dx.doi.org/10.1093/eurheartj/ehx502.P1621 96. Peng, J., Feng, B., Ren, W., Jiang, S., Wu, C., Hu, Z., & Xu, W. (2023). Incidence and risk factors of isolated calf muscular venous thrombosis after tibial plateau fractures surgery. *BMC Musculoskeletal Disorders, 24*(1), 625. 97. Peng, J., Wang, H., Zhang, L., & Lin, Z. (2021). Construction and efficiency analysis of prediction model for venous thromboembolism risk in the elderly after hip fracture. *Zhong nan da xue xue bao. Yi xue ban= Journal of Central South University. Medical Sciences, 46*(2), 142-148. 98. Peng, L., Bao, Q., Hong, X., Li, W., Zheng, Y., Zou, Z., . . . Huang, C. (2022). High level of neutrophil to lymphocyte ratio increases the risk of deep venous thrombosis in intensive care unit patients after oral cancer surgery: a retrospective study. *Annals of Translational Medicine, 10*(14). 99. Poredos, P., Jezovnik, M. K., Mavric, A., Leben, L., Mijovski, M. B., Maia, P., . . . Fareed, J. (2021). Time Course of Inflammatory and Procoagulant Markers in the Early Period After Total Hip Replacement. *Clinical and Applied Thrombosis/Hemostasis, 27*(no pagination). doi:https://dx.doi.org/10.1177/1076029620985941 100. Rinaldi, I., Hamonangan, R., Azizi, M. S., Cahyanur, R., Wirawan, F., Fatya, A. I., . . . Winston, K. (2021). Diagnostic Value of Neutrophil Lymphocyte Ratio and D-Dimer as Biological Markers of Deep Vein Thrombosis in Patients Presenting with Unilateral Limb Edema. *Journal of Blood Medicine, 12*, 313-325. doi:10.2147/jbm.S291226 101. Rinde, F. B., Fronas, S. G., Ghanima, W., Vik, A., Hansen, J.-B., & Brækkan, S. K. (2020). D-dimer as a stand-alone test to rule out deep vein thrombosis. *Thrombosis Research, 191*, 134-139. 102. Selvaggio, S., Brugaletta, G., Abate, A., Musso, C., Romano, M., Di Raimondo, D., . . . Santo Signorelli, S. (2023). Platelet‑to‑lymphocyte ratio, neutrophil‑to‑lymphocyte ratio and monocyte‑to‑HDL cholesterol ratio as helpful biomarkers for patients hospitalized for deep vein thrombosis. *International Journal of Molecular Medicine, 51*(6), 52. 103. Seo, W.-W., Park, M.-S., Kim, S. E., Lee, J.-H., Park, D.-G., Han, K.-R., . . . Hyon, M.-S. (2021). Neutrophil–lymphocyte ratio as a predictor of venous thromboembolism after total knee replacement. *The Journal of Knee Surgery, 34*(02), 171-177. 104. Shah, A., Rana, H., Rathod, C., & Sheth, A. (2020). Prevalence, Demographics and Risk Factors of Intracranial Stenosis in Ischemic Stroke Patients Admitted at a Teaching Government Hospital in Central Gujarat. *Journal of the Indian Medical Association, 118(11)*, 48-52. 105. Sheng, Q., Zhao, H., Wu, S., & Liu, R. (2020). Underlying factors relating to acute myocardial infarction for coronary artery ectasia patients. *Medicine (United States), 99(36)*, E21983. doi:https://dx.doi.org/10.1097/MD.0000000000021983 106. Soydal, C., Araz, M., Nak, D., Akkus, P., Baltacioglu, M. H., Bilgic, S., & Kucuk, N. O. (2020). Analysis of prognostic factors in patients receiving transarterial radioembolization for unresectable hepatocellular carcinoma. *Nuclear Medicine Communications, 41(1)*, 73-77. doi:https://dx.doi.org/10.1097/MNM.0000000000001122 107. Stubbs, M. J., Mouyis, M., & Thomas, M. (2018). Deep vein thrombosis. *BMJ, 360*, k351. doi:10.1136/bmj.k351 108. Tabakin, A. L., Stein, M. N., Anderson, C. B., Drake, C. G., & Singer, E. A. (2020). Cytoreductive nephrectomy for metastatic renal cell carcinoma, the ultimate urologic 'Choosing Wisely' campaign: a narrative review. *Translational Cancer Research, 9(11)*, 7337-7349. doi:https://dx.doi.org/10.21037/tcr-20-2343 109. Tekin, S. B., Bozgeyik, B., & Mert, A. (2022). Relationship between admission neutrophil/lymphocyte, thrombocyte/lymphocyte, and monocyte/lymphocyte ratios and 1-year mortality in geriatric hip fractures: Triple comparison. [Geriatrik kalca kiriklarinda giris notrofil-lenfosit, trombosit-lenfosit, monosit-lenfosit oranlari ile bir yillik mortalite arasindaki iliski: Uclu karsilastirma.]. *Ulusal Travma ve Acil Cerrahi Dergisi, 28(11)*, 1634-1640. doi:https://dx.doi.org/10.14744/tjtes.2021.94799 110. Tham, T., Rahman, L., Persaud, C., Olson, C., & Costantino, P. (2018). Venous thromboembolism risk in head and neck cancer: significance of the preoperative platelet-to-lymphocyte ratio. *Otolaryngology–Head and Neck Surgery, 159*(1), 85-91. 111. Tichelaar, Y. I., Kluin-Nelemans, H. J., & Meijer, K. (2012). Infections and inflammatory diseases as risk factors for venous thrombosis. A systematic review. *Thrombosis and Haemostasis, 107*(5), 827-837. doi:10.1160/th11-09-0611 112. Toker, S., Hak, D. J., & Morgan, S. J. (2011). Deep vein thrombosis prophylaxis in trauma patients. *Thrombosis, 2011*, 505373. doi:10.1155/2011/505373 113. Tong, Y., Ying, R., Niu, M., & Xu, L. (2024). Effect of venous foot pump intervention on prevention of venous thromboembolism in patients with major orthopedic surgery: a systematic review and meta-analysis. *Frontiers in Cardiovascular Medicine, 11*, 1408334. 114. Vitiello, R., Matrangolo, M. R., El Motassime, A., Perna, A., Cianni, L., Maccauro, G., & Ziranu, A. (2022). Three-Dimension-Printed Custom-Made Prosthetic Reconstructions in Bone Tumors: A Single Center Experience. *Current Oncology, 29(7)*, 4566-4577. doi:https://dx.doi.org/10.3390/curroncol29070361 115. Wang, T., Guo, J., Long, Y., Yin, Y., & Hou, Z. (2022). Risk factors for preoperative deep venous thrombosis in hip fracture patients: a meta-analysis. *Journal of Orthopaedics and Traumatology, 23*(1), 19. 116. Wang, T., Guo, J., Long, Y., Yin, Y., & Hou, Z. (2022). Risk factors for preoperative deep venous thrombosis in hip fracture patients: a meta-analysis. *Journal of Orthopaedics and Traumatology, 23*(1), 19. 117. Wang, Z., Pei, W., Chen, L., Ning, Y., & Luo, Y. (2020). Mean Platelet Volume/Platelet Count Ratio is Associated with Poor Clinical Outcome After Aneurysmal Subarachnoid Hemorrhage. *Journal of Stroke and Cerebrovascular Diseases, 29(11) (no pagination)*(105208). doi:https://dx.doi.org/10.1016/j.jstrokecerebrovasdis.2020.105208 118. Weitz, J. I., Fredenburgh, J. C., & Eikelboom, J. W. (2017). A Test in Context: D-Dimer. *Journal of the American College of Cardiology, 70*(19), 2411-2420. doi:10.1016/j.jacc.2017.09.024 119. Wu, J. X., Qing, J. H., Yao, Y., Chen, D. Y., & Jiang, Q. (2021). Performance of age-adjusted D-dimer values for predicting DVT before the knee and hip arthroplasty. *Journal of Orthopaedic Surgery and Research, 16*, 1-6. 120. Xia, Z. H., Chen, W. H., & Wang, Q. (2023). Risk factors for venous thromboembolism following surgical treatment of fractures: a systematic review and meta‐analysis. *International Wound Journal, 20*(4), 995-1007. 121. Xiong, X., & Cheng, B. (2023). Preoperative risk factors for deep vein thrombosis in knee osteoarthritis patients undergoing total knee arthroplasty. *Journal of Orthopaedic Science, 28*(1), 180-187. 122. Xu, J., Li, L., Fu, J., Xu, C., Ni, M., Chai, W., . . . Chen, J. (2022). Early Clinical and Radiographic Outcomes of Robot-Assisted Versus Conventional Manual Total Knee Arthroplasty: A Randomized Controlled Study. *Orthopaedic Surgery, 14(9)*, 1972-1980. doi:https://dx.doi.org/10.1111/os.13323 123. Xu, J., Lupu, F., & Esmon, C. T. (2010). Inflammation, innate immunity and blood coagulation. *Hamostaseologie, 30*(1), 5-6, 8-9. 124. Yamagata, K., Fukuzawa, S., Uchida, F., Ishibashi-Kanno, N., Yanagawa, T., & Bukawa, H. (2021). Is Preoperative plate-lymphocyte ratio a predictor of deep vein thrombosis in patients with oral cancer during surgery? *Journal of Oral and Maxillofacial Surgery, 79*(4), 914-924. 125. Yamagata, K., Fukuzawa, S., Uchida, F., Ishibashi-Kanno, N., Yanagawa, T., & Bukawa, H. (2021). Is Preoperative plate-lymphocyte ratio a predictor of deep vein thrombosis in patients with oral cancer during surgery? *Journal of Oral and Maxillofacial Surgery, 79*(4), 914-924. 126. Yao, C., Zhang, Z., Yao, Y., Xu, X., Jiang, Q., & Shi, D. (2018). Predictive value of neutrophil to lymphocyte ratio and platelet to lymphocyte ratio for acute deep vein thrombosis after total joint arthroplasty: a retrospective study. *Journal of Orthopaedic Surgery and Research, 13*, 1-5. 127. Yao, M. M., Lin, T. T., Shi, X., Chen, M. G., Wu, J. X., Zhao, Y. P., & Lin, B. S. (2024). Thrombosis density ratio can predict the occurrence of pulmonary embolism and post-thrombotic syndrome in lower-extremity deep vein thrombosis patients. *Clinical Hemorheology and Microcirculation, 86(4)*, 395-405. doi:https://dx.doi.org/10.3233/CH-231778 128. Zeng, G., Li, X., Li, W., Wen, Z., Wang, S., Zheng, S., . . . Sun, C. (2023). A nomogram model based on the combination of the systemic immune-inflammation index, body mass index, and neutrophil/lymphocyte ratio to predict the risk of preoperative deep venous thrombosis in elderly patients with intertrochanteric femoral fracture: a retrospective cohort study. *Journal of Orthopaedic Surgery and Research, 18*(1), 561. 129. Zeng, L., Cai, H., Qiu, A., Zhang, D., Lin, L., Lian, X., & Chen, M. (2023). Risk factors for rehospitalization within 90 days in patients with total joint replacement: A meta-analysis. *Medicine, 102*(45), e35743. doi:10.1097/md.0000000000035743 130. Zhang, J., Li, J., Zhao, K., Meng, H., Zhu, Y., Zhang, Y., & Chen, W. (2021). Post-operative deep vein thrombosis in patients over sixty years of age diagnosed with closed distal femur fractures undergoing open reduction internal fixation. *International Orthopaedics, 45*, 1615-1623. 131. Zhang, J., Zhao, K., Li, J., Meng, H., Zhu, Y., & Zhang, Y. (2020). Age over 65 years and high levels of C-reactive protein are associated with the risk of preoperative deep vein thrombosis following closed distal femur fractures: a prospective cohort study. *Journal of Orthopaedic Surgery and Research, 15*, 1-9. 132. Zhao, W., Zhao, J., Liu, T., Liu, Z., Liu, L., & Zhang, Y. (2022). Incidence and risk factors of preoperative deep venous thrombosis following pelvic and acetabular fractures: a retrospective case–control study. *Journal of Orthopaedic Surgery and Research, 17*(1), 77. 133. Zhou, D., Cheng, H., Chen, W., Li, Z. Y., & Xu, K. L. (2019). Relationship between Thrombotic Events and Peripheral Neutrophil-to-lymphocyte Ratio in Patients with Newly Diagnosed Essential Thrombocythemia. [Chinese]. *Zhongguo Shi Yan Xue Ye Xue Za Zhi. Journal of Experimental Hematology, 27(2)*, 534-538. doi:https://dx.doi.org/10.19746/j.cnki.issn.1009-2137.2019.02.036 134. Zhu, X., Yao, Y., Yao, C., & Jiang, Q. (2018). Predictive value of lymphocyte to monocyte ratio and monocyte to high-density lipoprotein ratio for acute deep vein thrombosis after total joint arthroplasty: a retrospective study. *Journal of Orthopaedic Surgery and Research, 13*, 1-7. 135. Zuo, J., & Hu, Y. (2020). Admission deep venous thrombosis of lower extremity after intertrochanteric fracture in the elderly: a retrospective cohort study. *Journal of Orthopaedic Surgery and Research, 15*(1), 549. doi:10.1186/s13018-020-02092-9 | |
| **Studies assessed for eligibility** | |
| 1. Diao, S., Li, J., Zhao, J., Wang, D., Wang, H., Xu, X., & Zhou, J. (2022). Risk factors and new inflammatory indicators of deep vein thrombosis after adult patella fractures. Frontiers in surgery, 9, 1028542. 2. Gao, Z., Zhao, K., Jin, L., Lian, X., Zhang, Z., Ma, L., & Hou, Z. (2023). Combination of neutrophil to lymphocyte ratio, platelet to lymphocyte ratio with plasma D-dimer level to improve the diagnosis of deep venous thrombosis (DVT) following ankle fracture. Journal of Orthopaedic Surgery and Research, 18(1), 362. 3. Liu, D., Zhu, Y., Chen, W., Li, J., Zhao, K., Zhang, J., . . . Zhang, Y. (2020). Relationship between the inflammation/immune indexes and deep venous thrombosis (DVT) incidence rate following tibial plateau fractures. Journal of Orthopaedic Surgery and Research, 15, 1-8. 4. Melinte, R. M., Arbănași, E. M., Blesneac, A., Zolog, D. N., Kaller, R., Mureșan, A. V., . . . Russu, E. (2022). Inflammatory biomarkers as prognostic factors of acute deep vein thrombosis following the total knee arthroplasty. Medicina, 58(10), 1502. 5. Niu, S., Pei, Y., Hu, X., Ding, D., & Jiang, G. (2022). Relationship between the neutrophil-to-lymphocyte ratio or platelet-to-lymphocyte ratio and deep venous thrombosis (DVT) following femoral neck fractures in the elderly. Frontiers in Surgery, 9, 1001432. 6. Peng, J., Wang, H., Zhang, L., & Lin, Z. (2021). Construction and efficiency analysis of prediction model for venous thromboembolism risk in the elderly after hip fracture. Zhong nan da xue xue bao. Yi xue ban= Journal of Central South University. Medical Sciences, 46(2), 142-148. 7. Seo, W.-W., Park, M.-S., Kim, S. E., Lee, J.-H., Park, D.-G., Han, K.-R., . . . Hyon, M.-S. (2021). Neutrophil–lymphocyte ratio as a predictor of venous thromboembolism after total knee replacement. The Journal of Knee Surgery, 34(02), 171-177. 8. Xiong, X., & Cheng, B. (2023). Preoperative risk factors for deep vein thrombosis in knee osteoarthritis patients undergoing total knee arthroplasty. Journal of Orthopaedic Science, 28(1), 180-187. 9. Yao, C., Zhang, Z., Yao, Y., Xu, X., Jiang, Q., & Shi, D. (2018). Predictive value of neutrophil to lymphocyte ratio and platelet to lymphocyte ratio for acute deep vein thrombosis after total joint arthroplasty: a retrospective study. Journal of Orthopaedic Surgery and Research, 13, 1-5. 10. Zeng, G., Li, X., Li, W., Wen, Z., Wang, S., Zheng, S., . . . Sun, C. (2023). A nomogram model based on the combination of the systemic immune-inflammation index, body mass index, and neutrophil/lymphocyte ratio to predict the risk of preoperative deep venous thrombosis in elderly patients with intertrochanteric femoral fracture: a retrospective cohort study. Journal of Orthopaedic Surgery and Research, 18(1), 561. 11. Bhat, T. M., Afari, M. E., & Garcia, L. A. (2016). Neutrophil lymphocyte ratio in peripheral vascular disease: a review. Expert Review of Cardiovascular Therapy, 14(7), 871-875. doi:https://dx.doi.org/10.1586/14779072.2016.1165091 12. Osunronbi, T., Olukoya, O., Jesuyajolu, D., Alare, K., Alemenzohu, H. O., Bello, R. O., . . . Sharma, H. (2024). The prognostic utility of neutrophil-lymphocyte ratio in spinal surgery: A systematic review and meta-analysis. Journal of Clinical Neuroscience, 121, 161-168. doi:https://dx.doi.org/10.1016/j.jocn.2024.02.021 13. Park, M. S., Seo, W. W., Kim, S. E., Lee, J. H., Park, D. G., Han, K. R., & Oh, D. J. (2017). Neutrophil lymphocyte ratio as a predictor of venous thromboembolism after total knee replacement. European Heart Journal, 38(Supplement 1), 348-349. doi:https://dx.doi.org/10.1093/eurheartj/ehx502.P1621. 14. Grimnes G, Horvei LD, Tichelaar V, Brækkan SK, Hansen JB. Neutrophil to lymphocyte ratio and future risk of venous thromboembolism and mortality: the Tromsø Study. Haematologica. 2016 Oct;101(10):e401-e404. 15. Kuplay, H., Erdoğan, S. B., Bastopcu, M., Arslanhan, G., Baykan, D. B., & Orhan, G. (2020). The neutrophil-lymphocyte ratio and the platelet-lymphocyte ratio correlate with thrombus burden in deep venous thrombosis. Journal of Vascular Surgery: Venous and Lymphatic Disorders, 8(3), 360-364. 16. Liu, H., Chen, X., Wang, Z., Liu, Y., & Liu, M. (2023). High systemic inflammation response index level is associated with an increased risk of lower extremity deep venous thrombosis: a large retrospective study. Annals of Medicine, 55(2), 2249018. 17. Ma, J., Qin, J., Hu, J., Shang, M., Zhou, Y., Liang, N., . . . Zhu, Y. (2020). Incidence and hematological biomarkers associated with preoperative deep venous thrombosis following foot fractures. Foot and Ankle International, 41(12), 1563-1570. 18. Peng, J., Feng, B., Ren, W., Jiang, S., Wu, C., Hu, Z., & Xu, W. (2023). Incidence and risk factors of isolated calf muscular venous thrombosis after tibial plateau fractures surgery. BMC Musculoskeletal Disorders, 24(1), 625. 19. Zhang, J., Li, J., Zhao, K., Meng, H., Zhu, Y., Zhang, Y., & Chen, W. (2021). Post-operative deep vein thrombosis in patients over sixty years of age diagnosed with closed distal femur fractures undergoing open reduction internal fixation. International Orthopaedics, 45, 1615-1623. 20. Zhang, J., Zhao, K., Li, J., Meng, H., Zhu, Y., & Zhang, Y. (2020). Age over 65 years and high levels of C-reactive protein are associated with the risk of preoperative deep vein thrombosis following closed distal femur fractures: a prospective cohort study. Journal of Orthopaedic Surgery and Research, 15, 1-9. | |
| **Reasons** | **Studies excluded after full-text review (n=10)** |
| Review article | 1. Bhat, T. M., Afari, M. E., & Garcia, L. A. (2016). Neutrophil lymphocyte ratio in peripheral vascular disease: a review. Expert Review of Cardiovascular Therapy, 14(7), 871-875. doi:https://dx.doi.org/10.1586/14779072.2016.1165091 2. Osunronbi, T., Olukoya, O., Jesuyajolu, D., Alare, K., Alemenzohu, H. O., Bello, R. O., . . . Sharma, H. (2024). The prognostic utility of neutrophil-lymphocyte ratio in spinal surgery: A systematic review and meta-analysis. Journal of Clinical Neuroscience, 121, 161-168. doi:https://dx.doi.org/10.1016/j.jocn.2024.02.021 |
| Abstract | 1. Park, M. S., Seo, W. W., Kim, S. E., Lee, J. H., Park, D. G., Han, K. R., & Oh, D. J. (2017). Neutrophil lymphocyte ratio as a predictor of venous thromboembolism after total knee replacement. European Heart Journal, 38(Supplement 1), 348-349. doi:https://dx.doi.org/10.1093/eurheartj/ehx502.P1621. 2. Grimnes G, Horvei LD, Tichelaar V, Brækkan SK, Hansen JB. Neutrophil to lymphocyte ratio and future risk of venous thromboembolism and mortality: the Tromsø Study. Haematologica. 2016 Oct;101(10):e401-e404. |
| No relevant outcome | 1. Kuplay, H., Erdoğan, S. B., Bastopcu, M., Arslanhan, G., Baykan, D. B., & Orhan, G. (2020). The neutrophil-lymphocyte ratio and the platelet-lymphocyte ratio correlate with thrombus burden in deep venous thrombosis. *Journal of Vascular Surgery: Venous and Lymphatic Disorders, 8*(3), 360-364. 2. Liu, H., Chen, X., Wang, Z., Liu, Y., & Liu, M. (2023). High systemic inflammation response index level is associated with an increased risk of lower extremity deep venous thrombosis: a large retrospective study. *Annals of Medicine, 55*(2), 2249018. 3. Ma, J., Qin, J., Hu, J., Shang, M., Zhou, Y., Liang, N., . . . Zhu, Y. (2020). Incidence and hematological biomarkers associated with preoperative deep venous thrombosis following foot fractures. *Foot and Ankle International, 41*(12), 1563-1570. 4. Peng, J., Feng, B., Ren, W., Jiang, S., Wu, C., Hu, Z., & Xu, W. (2023). Incidence and risk factors of isolated calf muscular venous thrombosis after tibial plateau fractures surgery. *BMC Musculoskeletal Disorders, 24*(1), 625. 5. Zhang, J., Li, J., Zhao, K., Meng, H., Zhu, Y., Zhang, Y., & Chen, W. (2021). Post-operative deep vein thrombosis in patients over sixty years of age diagnosed with closed distal femur fractures undergoing open reduction internal fixation. *International Orthopaedics, 45*, 1615-1623. 6. Zhang, J., Zhao, K., Li, J., Meng, H., Zhu, Y., & Zhang, Y. (2020). Age over 65 years and high levels of C-reactive protein are associated with the risk of preoperative deep vein thrombosis following closed distal femur fractures: a prospective cohort study. *Journal of Orthopaedic Surgery and Research, 15*, 1-9. |
