# Supplementary material for: Association between neutrophile-to-lymphocyte ratio and risk of deep vein thrombosis in patient receiving lower extremity orthopedic surgery: A meta-analysis
Source: PLoS One. 2025 Feb 24;20(2):e0319107. doi: 10.1371/journal.pone.0319107 (PMC11849845; doi:10.1371/journal.pone.0319107)
Supplement: S3 Table — (DOCX) [file pone.0319107.s003.docx]

**S3 Table.** Quality of studies based on Newcastle-Ottawa Scale scores

| Studies | Selection | Comparability | Outcome | Total score |
| --- | --- | --- | --- | --- |
| Diao 2022 | ★★★★ | ★ | ★★★ | 8 |
| Gao 2023 | ★★★ | ★ | ★★★ | 7 |
| Liu 2020 | ★★★★ | ★ | ★★★ | 8 |
| Melinte 2022 | ★★★ | ★ | ★★★ | 7 |
| Niu 2022 | ★★★★ | ★ | ★★★ | 8 |
| Peng 2021 | ★★★ | ★ | ★★★ | 7 |
| Seo 2021 | ★★★ | ★ | ★★★ | 7 |
| Xiong 2023 | ★★★★ | ★ | ★★★ | 8 |
| Yao 2018 | ★★★ | ★ | ★★★ | 7 |
| Zeng 2023 | ★★★★ | ★ | ★★★ | 8 |
