# Supplementary material for: Association between neutrophile-to-lymphocyte ratio and risk of deep vein thrombosis in patient receiving lower extremity orthopedic surgery: A meta-analysis
Source: PLoS One. 2025 Feb 24;20(2):e0319107. doi: 10.1371/journal.pone.0319107 (PMC11849845; doi:10.1371/journal.pone.0319107)
Supplement: S4 Table — (DOCX) [file pone.0319107.s004.docx]

**S4 Table.** Details regarding preoperative neutrophile-to-lymphocyte ratio (NLR) measurement

| Studies | Preoperative NLR measurement |
| --- | --- |
| Diao 2022 | Venous blood was collected from the elbow veins on an  empty stomach for the first time after admission. |
| Gao 2023 | Routine blood test was performed immediately after  Admission. |
| Liu 2020 | Biomarkers or biomarker-derived inflammatory/immune  indexes were obtained from hematologic tests carried  out after admission and before the definite operation. |
| Melinte 2022 | The patient blood test result was collected from the hospital’s computerized database. |
| Niu 2022 | Biomarkers or biomarker-derived inflammatory/immune indexes at admission or the first time after admission were selected for data analysis to eliminate their potential time dependent effect to the maximum extent. |
| Peng 2021 | The patient blood test result was collected from the hospital’s computerized database. |
| Seo 2021 | Biochemical tests were performed within 2weeks prior to TKR. |
| Xiong 2023 | Routine blood test was collected after admission. |
| Yao 2018 | The blood specimen was collected from the peripheral  venous before the operation and on the first morning  after the operation. |
| Zeng 2023 | Patients’ routine blood, hemostatic function, and other  serological test results acquired at emergency department or in one day after admission were collected. |
