# Supplementary material for: Association between neutrophile-to-lymphocyte ratio and risk of deep vein thrombosis in patient receiving lower extremity orthopedic surgery: A meta-analysis
Source: PLoS One. 2025 Feb 24;20(2):e0319107. doi: 10.1371/journal.pone.0319107 (PMC11849845; doi:10.1371/journal.pone.0319107)
Supplement: S5 Table — (DOCX) [file pone.0319107.s005.docx]

**S5 Table.** Raw data used in current meta-analysis (Name of data extractors: I-Wen Chen and Wei-Ting Wang; Date of data extraction: August 2024; All authors confirmed that the studies below were eligible to be included in the review)

| Studies | Outcomes |  | DVT | |  | Non-DVT | |
| --- | --- | --- | --- | --- | --- | --- | --- |
|  |  | Odds ratio | events | number |  | event | number |
| Diao 2022 | OR |  | 24 | 190 |  | 15 | 310 |
| Liu 2020 | OR |  | 94 | 432 |  | 98 | 747 |
| Gao 2023 | OR | 2.16 (1.34-3.49) |  |  |  |  |  |
| Melinte 2022 | OR | 10.15 (3.94-26.15) |  |  |  |  |  |
| Niu 2022 | OR | 0.7 (0.3-1.5) |  |  |  |  |  |
| Peng 2021 | OR | 2.003 (0.798-5.026) |  |  |  |  |  |
| Seo 2021 | OR | 1.95 (1.16-3.31) |  |  |  |  |  |
| Yao 2018 | OR | 1.11 (1.01-1.23) |  |  |  |  |  |
| Zeng 2023 | OR | 7.29 (1.53-34.64) |  |  |  |  |  |
|  |  |  |  |  |  |  |  |
|  |  | DVT | | | Non-DVT | | |
|  |  | mean | sd | n | mean | sd | n |
| Chen 2024 | mean | 10.7 | 6.8 | 72 | 9 | 5.9 | 170 |
| Gao 2023 | mean | 5.7 | 3.1 | 92 | 4.7 | 3.2 | 1011 |
| Melinte 2022 | mean | 5.54 | 2.02 | 28 | 3.05 | 1.25 | 245 |
| Peng 2021 | mean | 5.56 | 2.81 | 52 | 3.98 | 1.65 | 52 |
| Seo 2021 | mean | 2.57 | 1.59 | 102 | 2.11 | 1.1 | 162 |
| Xiong 2023 | mean | 2.10 | 1.10 | 40 | 1.84 | 0.77 | 544 |
| Yao 2018 | mean | 2.6 | 2.3 | 120 | 2.3 | 1.7 | 653 |
|  |  |  |  |  |  |  |  |
|  |  |  |  |  |  |  |  |
